# Analysis of proteomes released from in vitro cultured eight *Clostridium difficile* PCR ribotypes revealed specific expression in PCR ribotypes 027 and 176 confirming their genetic relatedness and clinical importance at the proteomic level

**DOI:** 10.1186/s13099-017-0194-9

**Published:** 2017-08-14

**Authors:** Jiri Dresler, Marcela Krutova, Alena Fucikova, Jana Klimentova, Veronika Hruzova, Miloslava Duracova, Katerina Houdkova, Barbora Salovska, Jana Matejkova, Martin Hubalek, Petr Pajer, Libor Pisa, Otakar Nyc

**Affiliations:** 1 Military Health Institute, Military Medical Agency, Tychonova 1, Prague, Czech Republic; 2 Department of Medical Microbiology, 2nd Faculty of Medicine, Charles University in Prague and Motol University Hospital, Prague, Czech Republic; 3 Faculty of Military Health Sciences, UoD, Hradec Kralove, Czech Republic; 4 Institute of Organic Chemistry and Biochemistry, Academy of Science, Prague, Czech Republic

**Keywords:** *Clostridium difficile*, Label-free quantification, Proteome, PCR ribotype 027, PCR ribotype 176, Binary toxin, Toxins A/B, Flagellins

## Abstract

**Background:**

*Clostridium difficile* is the causative agent of *C*. *difficile* infection (CDI) that could be manifested by diarrhea, pseudomembranous colitis or life-threatening toxic megacolon. The spread of certain strains represents a significant economic burden for health-care. The epidemic successful strains are also associated with severe clinical features of CDI. Therefore, a proteomic study has been conducted that comprises proteomes released from in vitro cultured panel of eight different PCR ribotypes (RTs) and employs the combination of shotgun proteomics and label-free quantification (LFQ) approach.

**Results:**

The comparative semi-quantitative analyses enabled investigation of a total of 662 proteins. Both hierarchical clustering and principal component analysis (PCA) created eight distinctive groups. From these quantifiable proteins, 27 were significantly increased in functional annotations. Among them, several known factors connected with virulence were identified, such as toxin A, B, binary toxin, flagellar proteins, and proteins associated with Pro–Pro endopeptidase (PPEP-1) functional complex. Comparative analysis of protein expression showed a higher expression or unique expression of proteins linked to pathogenicity or iron metabolism in RTs 027 and 176 supporting their genetic relatedness and clinical importance at the proteomic level. Moreover, the absence of putative nitroreductase and the abundance of the Abc-type fe3+ transport system protein were observed as biomarkers for the RTs possessing binary toxin genes (027, 176 and 078). Higher expression of selected flagellar proteins clearly distinguished RTs 027, 176, 005 and 012, confirming the pathogenic role of the assembly in CDI. Finally, the histidine synthesis pathway regulating protein complex HisG/HisZ was observed only in isolates possessing the genes for toxin A and B.

**Conclusions:**

This study showed the applicability of the LFQ approach and provided the first semi-quantitative insight into the proteomes released from in vitro cultured panel of eight RTs. The observed differences pointed to a new direction for studies focused on the elucidation of the mechanisms underlining the CDI nature.

**Electronic supplementary material:**

The online version of this article (doi:10.1186/s13099-017-0194-9) contains supplementary material, which is available to authorized users.

## Background

*Clostridium difficile* is a ubiquitous Gram positive spore-forming anaerobic bacterium. Toxin-producing strains of *C. difficile* can cause infection (CDI) manifested by diarrhea, pseudomembranous colitis, or severe form, toxic megacolon. The spread of certain *C. difficile* PCR ribotypes in health-care setting has been reported and the global increasing trend of CDI incidence is unfavorable [1].

Several well-studied major virulence factors, such as *C. difficile* toxins (toxin A—TcdA and toxin B—TcdB) that are influential in severity of the CDI are at the focus of current research [2]. The different levels of toxin expression in vivo was reported [1] as well as the connection between the expression of toxins and flagellar proteins, which are involved in motility and gut colonization [3].

Previous proteomic studies were based on investigation of either the whole cell lysates [4, 5], or culture supernatants representing possible secretome in vitro [2, 6]. For comparative proteomic analyses difference gel electrophoresis (DIGE) [4] or isotopic labelling of selected proteins [7] were used.

The aforementioned studies enabled the analyses of the large toxins TcdA and TcdB [2] and also highlighted the role of proteins involved in the adhesion and cell surface composition. The differences in the level of expression of proteins including adhesins, S-layer proteins, cell wall proteins as well as a number of S layer protein paralogues [4–6] and other potential virulence factors were identified and proposed to play a role in the virulence characteristics of individual isolates.

This proteomic study used the combination of shotgun proteomics—to attain proteomic profile—and the label-free quantification (LFQ) approach, for semi-quantitative analysis. Shotgun proteomics which uses a high-resolution tandem mass spectrometry enables the analysis of hundreds of proteins in a cost-effective manner.

In spite of its accuracy and sensitivity, the major drawbacks of the gel-based approach are that relatively high amount of the protein samples is needed and its labor-intensiveness. Moreover, labelling-based techniques are limited by the need of expensive consumables, an inability to add further samples into the experiment and a limited number of compared groups [8]. On the contrary, LFQ does not require any labelling step in the sample preparation workflow and relies only on spectral counting or MS^1^ intensity of the quantified feature. That the use of labels is unnecessary provides this method with several attractive benefits: the implementation cost is low; the lack of additional steps reduces undesirable biases in the analyses; and the number of treatment conditions and sample replicates is basically unrestrained. Collectively, these features allow for flexibility in experimental design. Furthermore, recent developments in label-free quantification software have increased the robustness of label-free quantitation workflows by introducing sophisticated normalization and feature alignment algorithms [9]. In the bacterial proteomics, LFQ has been recently applied to the comparison of lysates [10] the relative abundance of ribosomal proteins in *Pseudomonas aeruginosa* [11] and to phosphoproteomes of *Bacillus subtilis* [12].

For the current proteomic comparative analysis, we decided to analyze proteomes released from in vitro cultured panel for the following reasons: (i) the released proteins are of high relevance for *Clostridia* pathogenicity and virulence; (ii) the complexity of the released fraction is much lower than that of cellular proteome and therefore it is more amenable for the scope of our analysis.

The panel of eight *C. difficile* isolates for the study was selected from the Czech *C. difficile* strain collection [13]. RTs 001 and 176 belong to the predominant RTs in the Czech Republic (26.7 and 20.7%, respectively), followed by RTs 014 and 012 (8.0 and 5.8%, respectively), [13]. The occurrence of RTs 027 and 078 in the Czech Republic is rare (0.2, 1.6%, respectively) [13], however, these RTs were suggested as being “hypervirulent” [14, 15]. Moreover, a higher expression of TcdA and TcdB in RT 027 was previously observed [1]. RTs 027 (19%), 001 (11%) and 014 (10% together with RT 020) belong to the most frequently found RTs in Europe [16]. The protein expression profile of RT 005 together with RTs 001 and 027 was studied previously [4]. RT 010 was included in the study as a “negative control” due to the absence of a pathogenicity locus.

## Methods

### *Clostridium difficile* isolates

Eight well characterized clinical isolates of *C. difficile* were selected from the Czech National *C. difficile* strain collection (Table 1) [13].

**Table 1:** Characterisation of *C. difficile* isolates in the study

Nr.	Isolate number^a^	Year of isolation	Patient age^b^	Sex	RT	ST	Clade	Presence of toxin genes	*tcdC truncation*
1	2063	2015	82	M	001	3	1	*tcdA*, *tcdB*	No
2	2023	2015	73	M	005	6	1	*tcdA*, *tcdB*	No
3	1107	2014	25	F	010	15	1	Non-toxigenic	NA
4	2006	2015	74	M	012	54	1	*tcdA*, *tcdB*	No
5	1120	2014	33	F	014	2	1	*tcdA*, *tcdB*	No
6	854	2014	35	M	027	1	2	*tcdA*, *tcdB*, *cdtA, cdtB*	Δ117
7	2004	2015	54	M	078	11	5	*tcdA*, *tcdB*, *cdtA, cdtB*	C184T
8	2062	2015	84	F	176	1	2	*tcdA*, *tcdB*, *cdtA, cdtB*	Δ117

^a^Number in the Czech national *C. difficile* strain collection

^b^Age of the patient at the time of isolation, RT-PCR-ribotype, ST-sequence type, tcdC truncation—single nucleotide polymorphism (SNP) resulting in TcdC protein truncation

Seven *C. difficile* isolates were cultured from diarrheal glutamate dehydrogenase (GDH) and toxin A/B positive stool samples of hospitalized patients with CDI. One *C. difficile* isolate (RT 010, non-toxigenic) was cultured from diarrheal GDH positive and toxin A/B negative stool sample of patient with Candida-acquired diarrhea. All isolates were sensitive to metronidazole (MTZ).

### Culture of *C. difficile* isolates and supernatant precipitation

*Clostridium difficile* isolates were recovered from the frozen stocks by inoculating on the Schaedler Anaerobe Agar CM0437 (Oxoid) and cultured for 48 h at 37 °C under anaerobic conditions. Toxin production of all strains in the study was confirmed using commercial immunochromatographic assay (Vidia, Czech Republic) for the detection of free toxins A and B in the stool samples when the bacterial suspension was investigated as a stool sample. The bacterial mass was resuspended in Thioglycolate medium USP (Oxoid) and the number of bacteria (CFU) was analyzed via optical density (OD) analysis at 595 nm (Multiskan Spectrum plate reader, Thermo Fisher Scientific), considering that OD 1 in 1 mL of Thioglycolate medium corresponds to 2.4 × 10^6^ CFU. Later, 9 mL of Thioglycolate medium was inoculated in triplicate for each representative strain to OD 1.99 and cultivated for 5 days at 37 °C under anaerobic conditions. OD was also measured at the end of the cultivation and reached comparable values among the cultures (Additional file 1: Table S1). Capillary electrophoresis ribotyping of *C. difficile* isolates was performed, using primers described elsewhere [17], before resuspension in Thioglycolate medium USP and after 5 days of culture before proteomic analysis.

### Sample preparation

Following the pelleting of bacterial cells by centrifugation (18,000*g*/20 min) to remove all bacterial cells from the proteomes released from in vitro cultures, the pH of the supernatants was adjusted to 3.5 with 3 M sulfuric acid (Sigma-Aldrich). After an overnight precipitation at 4 °C, the pellets were recovered by centrifugation (18,000*g*/20 min). Because of the potential of interfering substances in the supernatant, the sample preparation workflow was applied and based on FASP (Filter aided sample preparation—FASP) [18]. Pellets were resuspended in 100 mM ammonium bicarbonate (Sigma-Aldrich), and proteins were quantified by bicinchoninic acid assay, (QuantiPro™ BCA Assay Kit, Sigma-Aldrich), [19]. Data are available in the Additional file 1: Table S1. Resuspended pellets were transferred onto Amicon^®^ Ultra—10 kDa filters (Millipore) and washed twice by 100 mM ammonium bicarbonate. Subsequently, the samples were denatured by 8 M guanidinium chloride (Sigma-Aldrich), reduced with 100 mM Tris (2-carboxyethyl) phosphine hydrochloride (TCEP, Sigma-Aldrich) and alkylated with 300 mM iodoacetamide (Sigma-Aldrich). Finally, the samples were digested with 2 μg of sequencing grade trypsin (Promega) overnight at 37 °C. Empore™ SPE Cartridges, C18, standard density, bed I.D. 4 mm (Sigma-Aldrich) were used to desalt peptide mixtures before drying to completion in a speed-vac. Before the mass spectrometry analysis, the samples were resuspended in 30 μL of 2% acetonitrile (ACN)/0.1% trifluoroacteic acid. The samples were furthered analyzed by LC–MS/MS techniques involving targeted mass spectrometry and LFQ. Subcellular localization of the *proteins released* from in vitro cultured panel was evaluated by bioinformatic tools. Detailed description of procedure is described in the Additional file 1.

## Results

**Fig.1:**
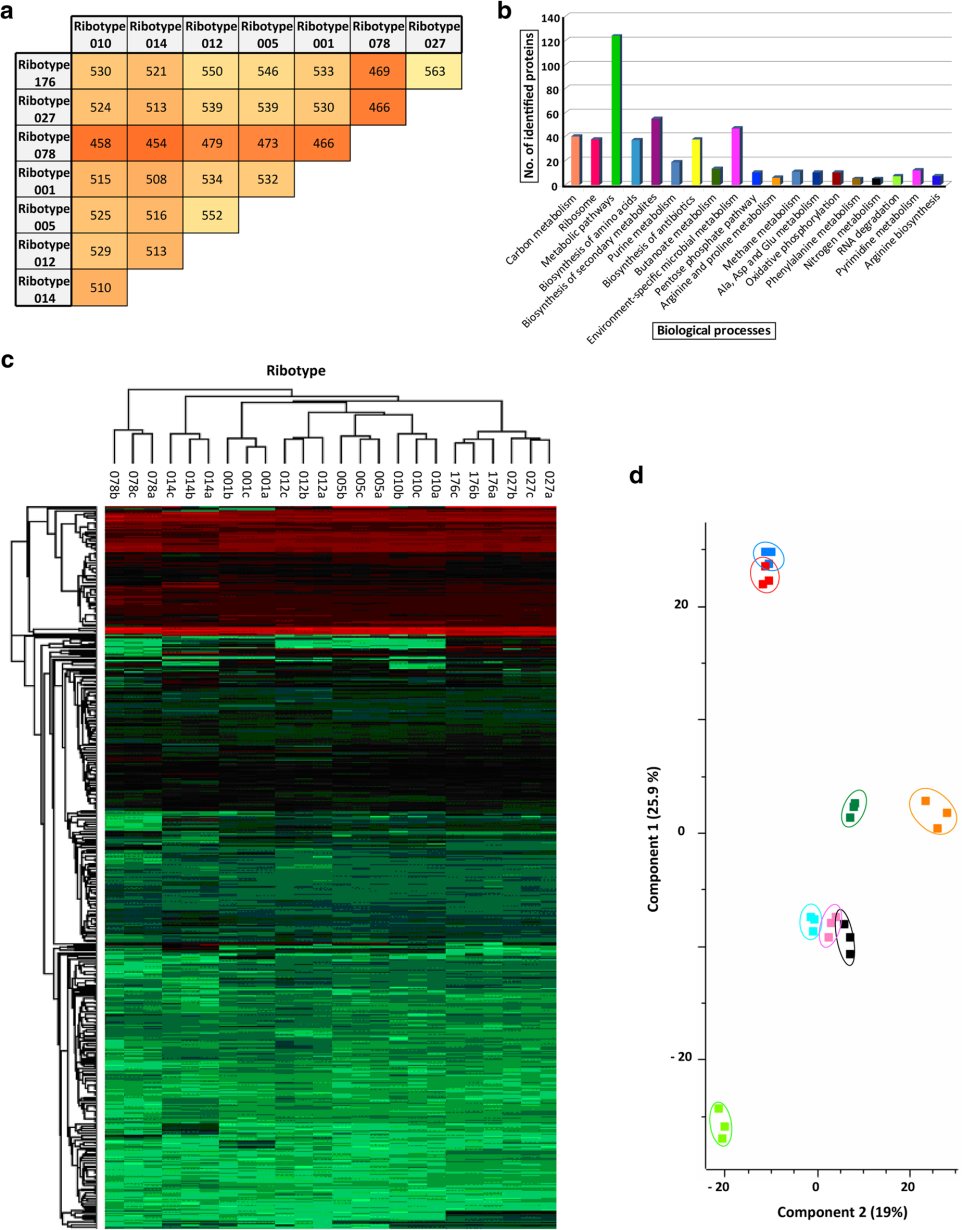
The overview of *C. difficile* proteomes. **a** The number of proteins that any pair of proteomes share showing overlapping identifications. **b** The mapping of the biological processes in KEGG annotated to 40.1% of quantifiable proteins in DAVID. The data were extracted from Additional file 1: Table S3. **c** Hierarchical clustering of the median protein expression values based on label-free proteome quantification. LFQ values versus biological triplicates (designated as **a**, **b** and **c**) from eight isolates of analyzed PCR ribotypes. The *color code* indicates the LFQ values abundance of the 662 quantifiable proteins (*red* most abundant; *green* least abundant). **d** Principal component analysis (PCA) of the LFQ intensities obtained from biological triplicate of each representative *C. difficile* isolate

Using the LFQ approach, a total of 662 quantifiable proteins were analyzed (Additional file 1: Table S3). The observed quantities of the proteins are depicted as log_2_ transformation of LFQ intensities [9]. The values ranging from 22 to 34 reflect the dynamic range of the mass spectrometry based workflow. The LFQ intensities below this value are considered as non-analyzable by the implemented qualitative test [20]. The most shared proteins were observed in RTs 027 and 176 (n = 563) and the lowest rate revealed RT 078 compared to all RTs in the study (n = 454–479), see Fig. 1a. Pathway mapping in KEGG was done using the DAVID classification tool [21] against *C. difficile* strain 630, and several biological processes were annotated to 40.1% of the quantifiable proteins (Fig. 1b).

### Comparison of proteomes of individual RTs

To assess the applicability of the LFQ approach, we examined the similarity of individual proteomes using hierarchical clustering and principal component analysis. Unsupervised cluster analysis of protein expression profiles was performed using Euclidean distances. Statistical procedures were performed using the computational platform Perseus [9]. Both hierarchical clustering (Fig. 1c) and PCA (Fig. 1d) generated eight distinctive groups encompassing each biological triplicate of analyzed RT representatives and showed the applicability of this workflow. For example, RTs 027 and 176 nearly co-cluster, on the contrary proteomes from RT 078 created a distinctive group.

#### The selection of proteins significantly increased in functional annotations

The selected proteins were chosen as candidates passing through ANOVA statistical test and subsequent Fisher exact test for increase in functional annotations. The details of the procedures are described below. The imputation of missing values from a normal distribution (Gaussian distribution width 0.3 SD and down-shift 1.8 SD of the original data) was performed, and proteins were annotated by Gene Ontology (GO) terms and UniProt keywords for the strain R20291 or for the strain 630 (downloaded on December 19th 2015). Furthermore, ANOVA (permutation-based FDR 5%, S0 = 0) was used to identify significant differences in protein expression between the RTs. Only ANOVA-significant hits were used for subsequent hierarchical clustering using Euclidean distances to group proteins with similar expression profiles. Finally, Fisher exact test at 2% Benjamini–Hochberg FDR was applied to determine significantly overrepresented functional annotations for each one of the identified clusters. In total, 27 proteins were found to be significantly increased in functional annotations. The MS data of these proteins are shown in the Additional file 1: Table S4; the results for proteins significantly increased in functional annotations are shown in Table 2).

**Table 2:** LFQ analysis of proteins significantly increased in functional annotations depicted as log2 median protein LFQ intensities for each PCR-ribotype

Protein IDs	Gene	Other name	LFQ intensities (medians)							
			PCR ribotype 010	PCR ribotype 014	PCR ribotype 012	PCR ribotype 005	PCR ribotype 001	PCR ribotype 078	PCR ribotype 027	PCR ribotype 176
Proteins connected with pathogenicity										
C9YJ37	tcdA		NaN	NaN	23.9406471252441	21.0392570495605	NaN	22.6982688903809	29.6992073059082	30.8379325866699
C9YJ35	tcdB		NaN	NaN	NaN	NaN	NaN	25.519832611084	28.9616012573242	29.5168876647949
C9YPH7	CDR20291_2491	Binary toxin A	NaN	NaN	NaN	NaN	NaN	24.3312873840332	26.6904582977295	26.4365386962891
C9YPH8	CDR20291_2492	Binary toxin B	NaN	NaN	NaN	NaN	NaN	23.4304580688477	28.8938579559326	28.5896377563477
C9YK92	CDR20291_0993	Sigma-54 dependent regulatory protein	NaN	NaN	NaN	27.0586080551147	NaN	NaN	27.1893329620361	26.7876834869385
C9YLG4	CDR20291_1416	Nucleic acid zinc binding protein	NaN	NaN	NaN	NaN	NaN	NaN	25.9671478271484	26.3175354003906
C9YLL2	CDR20291_1464	Cell wall binding repeat 2 family	NaN	NaN	NaN	NaN	NaN	NaN	26.3065376281738	25.8410053253174
Proteins connected with nitric group reduction and Iron metabolism										
C9YRI2	CDR20291_3199	Putative nitroreductase	27.0130786895752	26.4193916320801	26.5232486724854	26.8069801330566	26.496114730835	NaN	NaN	NaN
C9YQW5	CDR20291_2983	Abc-type Fe3 + transport protein	NaN	NaN	NaN	NaN	NaN	26.9341583251953	26.2506980895996	26.5248928070068
Proteins involved in assembly of *C. difficile* flagellum										
C9YI65	flgE		NaN	26.9150	NaN	27.8812	NaN	NaN	28.3666	28.8633
C9YI79	flgG		NaN	26.0100	NaN	26.5176	NaN	NaN	27.1453	27.7334
C9YI39	flgK		NaN	25.8299	NaN	27.1115	NaN	20.8008	27.4089	27.8577
C9YI40	flgL		NaN	26.9301	NaN	28.2372	NaN	NaN	28.8744	29.2726
C9YI47	fliC		22.6972	31.3676	24.8220	32.0720	23.0162	24.4515	33.4973	33.4187
C9YI45	fliD		NaN	28.5824	NaN	29.5971	NaN	24.4789	30.8483	31.3318
C9YI63	fliK		NaN	24.3520	NaN	24.2037	NaN	NaN	25.4507	25.9487
C9YI80	CDR20291_0273	Flagellar basal body protein	NaN	25.7288	NaN	25.6922	NaN	NaN	27.1761	27.5685
C9YI56	flgC		NaN	23.3975	NaN	21.6154	NaN	NaN	23.3294	24.2061
C9YI37	flgM		NaN	22.7971	NaN	23.0258	NaN	NaN	24.9594	24.9965
C9YI69	fliL		NaN	NaN	NaN	24.3746	NaN	NaN	24.1803	24.3687
C9YI57	fliE		NaN	NaN	NaN	NaN	NaN	NaN	24.7266	25.1837
C9YI34	CDR20291_0227	Glycosyltransferase	NaN	23.5203	NaN	23.5857	NaN	NaN	25.4568	25.7181
Pro–Pro endopeptidase, PPEP-1										
Q183R7	CDR20291_2721	The Pro–Pro endopeptidase PPEP-1	30.2371	29.8636	29.8793	29.7003	29.8969	29.2947	28.2033	28.3333
Q183R6	CD2831/Q183R6		NaN	NaN	NaN	NaN	NaN	24.6508	NaN	NaN
Q17ZZ0	CD3246/Q17ZZ0		NaN	NaN	NaN	NaN	NaN	26.6933	NaN	NaN
Proteins involved in histidine pathway metabolism										
C9YLE4	hisZ		NaN	27.7653	27.1694	28.0111	28.4014	27.1528	27.5777	27.9507
C9YLE5	hisG		NaN	26.6600	26.5274	27.2147	27.6760	26.3144	26.7599	27.0261

NaN represent values not detected in the particular isolates

#### Bioinformatic analysis of subcellular localization of the proteins released from in vitro cultured panel

To identify the subcellular localization of the proteins released from in vitro cultured panel, the bioinformatic analyses focused on Sec pathway and alternative secretion modes markers were performed. The sequences of all identified proteins were processed with SignalP 4.1 (http://www.cbs.dtu.dk/services/SignalP/) [22]. The secretion type of protein identified was predicted as “classical” if a signal peptide was identified with *Signal P Score* > 0.5. Furthermore, the Secretome P 2.0 tool (http://www.cbs.dtu.dk/services/SecretomeP/) [23] was employed. Using default parameters for Gram-positive bacteria and Secretome P Score > 0.5, proteins were predicted as “alternatively secreted”. A majority of the proteins found to be significantly increased in functional annotations were predicted to be secreted via sec-dependent secretion pathway or via an alternative secretion system (see Additional file 1: Table S4—proteins designated as SigP for classical and SecP for alternative secretion) proposing the overlapping of the supernatant proteome and the secretome. However, Zinc binding protein (C9YLG4), putative nitroreductase C9YRI2, FliC, FliL, HisG, and HisZ were not predicted to be secreted via any secretion system and are reportedly localized intracellularly.

### Proteins connected with pathogenicity

Using the targeted mass spectrometry, TcdA was detected as highly produced in RTs 027 and 176. Lower quantities of TcdA were observed in RTs 005 and 012. The protein was not detected in RTs 010, 078, and 001. TcdB was detected exclusively in RTs 027 and 176. Employing heavy labeled peptides and targeted mass spectrometry for TcdA and TcdB, the analysis confirmed these results (Additional file 1: Table S5, Figure S2) and approved the LFQ approach relevancy. The expression differences of selected proteins connected with pathogenicity are depicted in Table 2.

CdtA and CdtB/(binary toxin) were observed in the secreted fractions of RTs 027, 078 and 176, and in high levels in RTs 027 and 176.

The expression of Sigma-54 dependent regulatory protein (C9YK92), responsible for creating swift and precise responses to environmental change [24], was observed among RTs 027, 176, and 005. Furthermore, the high expression of nucleic acid zinc binding protein (C9YLG4) and the cell surface, putative penicillin binding protein, which belongs to the cell wall binding repeat 2 family (C9YLL2), were observed exclusively in RTs 027 and 176.

### Proteins connected with nitric group reduction and iron metabolism

The nitroreductase C9YRI2, an enzyme putatively involved in the activation of the MTZ, was expressed at similar levels in RTs 001, 005, 010, 012, and 014, but was not detected in RTs 027, 176, and 078. In contrast, Abc-type Fe3+ transport system periplasmic component-like protein C9YQW5 was found in high levels only in RTs 027, 176, and 078.

### Proteins involved in the assembly of *C. difficile* flagellum

A higher expression of flagellar proteins FlgE, G, K, L, Fli C, D, K, and flagellar basal body protein C9YI80 were observed among RTs 027, 176, 005, and 014 with the exception of FlgK and FliD quantifiable also in RT 078. Moreover, FlgC levels were increased only in RTs 027, 176, and 014, whilst FlgM and FliL were found to be increased in RTs 027, 176, and 005 (Table 2). Interestingly, FliE was expressed only in RTs 027 and 176, and glycosyltransferase C9YI34, which is involved in the post-translational modification of flagellum, was expressed in RTs 027, 176, 005, and 014.

### Pro–Pro endopeptidase (PPEP-1)

The Pro–Pro endopeptidase (PPEP-1) expression was observed in all RTs without relevant differences (Table 2). In addition, the search for substrates of PPEP-1 was also performed. Since PPEP-1 gene is probably not present in R20291 genome/proteome, the reference strain 630 [25, 26] was employed. The substrates CD2831/Q183R6 and CD3246/Q17ZZ0 were detected only in the replicates of RT 078 isolate (Table 2).

### Proteins involved in histidine pathway metabolism

HisG (C9YLE5) a HisZ (C9YLE4) were not detected in non-toxigenic RT 010 (Table 2). In other RTs, the expression of these proteins was observed in high levels but without distinct differences.

## Discussion

In this study, we used a combination of MS-based shotgun proteomics and the LFQ approach. Previously published qualitative studies involved less than three *C. difficile* representatives [4, 6]. In contrast, the unlimited number of compared groups in the LFQ technique enabled the semi-quantitative investigation of the proteomes released from in vitro cultured panel of eight *C. difficile* isolates and the applicability of this workflow was shown by hierarchical clustering and PCA; both methods generated eight distinctive groups encompassing each biological triplicate of RTs analyzed (see Fig. 1).

RTs 027 and 176 revealed higher expression of proteins connected to pathogenicity (TcdA, TcdB, CdtA, CdtB, sigma-54 dependent regulatory protein, nucleic acid zinc binding protein, the cell wall binding repeat 2 family), which confirms their clinical importance [1, 27] and evolutionary relationship at the proteomic level. Finally, RT 078, also referred to as hyper virulent [15], showed measurable levels of toxins but in lower levels compared to RTs 027 and 176.

Recent studies reported that the orphan response regulator CdtR enhances production not only of CDT from the same locus (CDT locus), but also of TcdA and TcdB from the Pathogenicity Locus. It was confirmed in two RT 027 human strains and also supported in the animal model. Contrary to that, in RT 078 strain where *cdtR* is a pseudogene, and in RT 012 strain where *ctdA/B* are pseudogenes, the function of CdtR was not proven [28].

The inability to detect the large *C. difficile* toxins in RTs 014 and 001 in this proteomic study, in spite of the positivity in the immunochromatographic assay, could be caused by the generally lower sensitivity of even high-resolution tandem mass spectrometry compared to tests based on antibodies (the detection limits of immunochromatographic assay used here was 12.5 ng/mL). These findings are in agreement with previously published studies showing that very low levels of toxins produced in vitro by toxigenic strains are hardly detectable by mass spectrometry [2], especially in the non-hypervirulent representatives of *C. difficile* RTs [4].

RTs 027, 176, and 078 also showed higher expression of Abc-type Fe3+ transport system periplasmic component-like protein C9YQW5. The iron represents a crucial nutrition factor and the competition over its bioavailability plays an essential role within complex microbial communities as well as between bacterial pathogens and their eukaryotic hosts [29]. The pathways of iron inside the cell could involve high-affinity iron chelators known as siderophores translocated by specific ABC transporters [30]. On the other hand, high levels of intracellular iron can increase oxidative damage and therefore, the expression of iron acquisition mechanisms are tightly controlled by transcriptional regulators [31].

The homologues of C9YQW5 are present only in the UniProt proteomes of RTs also expressing the binary toxins. This could indicate the presence of a specific mechanism that is responsible for the iron uptake in RTs possessing binary toxin genes. However, the lack of other differentially expressed proteins participating in the metabolism of the iron does not support the hypothesis of increased ability of the iron uptake in these RTs.

Interestingly, no expression of C9YRI2, a putative nitroreductase, was observed in RTs 027, 176, and 078. However, all isolates involved in this study were susceptible to metronidazole (MTZ). Based on the reference proteomes, the genes for the homologs of nitroreductase C9YRI2 have been reported in RT 027 [25, 26] and RT 078 [15] and therefore, the probable absence of expression among these RTs may be imposed by some regulatory mechanism. For that reason, the effect of the reduced levels of C9YRI2 is probably compensated by other unidentified mechanism of activation of the prodrug. This hypothesis is supported by the study that compared MTZ resistant and susceptible North American pulse-field type 1, PCR ribotype 027 (NAP1/027) isolates, where no changes in this protein were observed [32]. Thus, the absence of expression of the protein C9YRI2 probably does not play a crucial role in MTZ resistance.

Despite the shared presence of genes for large and binary toxins, the RTs 027, 176, and 078 represent clearly distinctive entities based on their proteome profiles. The only common characteristics observed are changed expressions of Abc-type Fe3+ transport system protein C9YQW5 and the putative nitroreductase C9YRI2. In spite of the findings in this study, the comparative proteomic study on NAP1/027 clinical isolates resistant to MTZ showed upregulation of ferric uptake regulator (Fur) [32], but the connection between iron uptake regulation and MTZ resistance was not confirmed.

The proteins involved in the assembly of *C. difficile* flagellum and Pro–Pro endopeptidase were present among other proteins revealing higher expression. The quantification of the proteins involved in the assembly of *C. difficile* flagellum pointed toward RTs 027 and 176, the main proteins constituting hook–basal-body complex and the rotating filament were observed as overexpressed. The discriminatory proteins with expression characteristic only for RTs 027 and 176 involved FliE protein and glycosyltransferase C9YI34. FliE participates in the normal export of other substrates. However, a very low basal level of export function was previously described even in the absence of FliE. This argues against a vital role for FliE in export and proposes the primary role of FliE as a structural adapter between the annular symmetry of the membrane and supramembrane ring and the helical symmetry of the rod and all subsequent axial structures [33].

A homologue of glycosyltransferase C9YI34 (CD0240 in *C. difficile* 630) was proven to be involved in the glycosylation process. Inactivation of CD0240 led to loss of the surface-associated flagellin protein and rendered the strain non-motile. However, the strain still produced truncated polymerized flagella filaments [34, 35]. In our study, this protein was also observed in RTs 014 and 005. However, the expression levels were lower. Thus, flagellin glycosylation was confirmed to be important in *C. difficile* flagellum assembly and virulence.

Regarding RT 078, the absence of differential expression of most proteins involved in the assembly of *C. difficile* flagellum (with exception of FlgK and FliD) could be explained by previously published genomic study which confirmed the complete loss of the F3 flagellar region while retaining the F1 region (containing *fliK* and *fliD* genes). This has been corroborated using microarray data from phylogenetic studies [25, 26]. The low protein expression of the FliC, FliD and a putative glycosyltransferase (in comparison with RTs 027 and 176), is in agreement with the studies on non-flagellated *C. difficile* serotypes retaining transcription of *fliC* and *fliD* genes reporting the absence of its protein products [36, 37].

The question of a correlation between particular flagellins and toxin levels among RTs 027, 176, and 005 could be raised. However, the lower levels of TcdA and TcdB in RT 014 and the inability to detect the flagellar proteins by the mass spectrometry proposes the greater complexity of the of the *C. difficile* virulence factors.

The Pro–Pro endopeptidase, PPEP-1, CD2830 alias C9YQ56 in strain R20191 reference proteome, was analysed as a highly active secreted metalloprotease and potential marker of virulence. The identification of two *C. difficile* LPXTG surface proteins CD2831 and CD3246 as highly efficient substrates for PPEP-1 indicated a role for this enzyme in bacterial motility [38]. However, the decreased tendency of the LFQ intensities of PPEP-1 toward RTs 027 and 176 was observed in this study. Clearly, the question of the functionality of these findings remains to be addressed.

Regarding the substrates, PPEP-1 knockout strain was demonstrated to have higher affinity for collagen type I with attenuated virulence in hamsters due to the cleavage of collagen binding protein CD2831/C9YQ57 [39], and this protein was described to be completely released from the cells [40]. In our study the later substrate, CD3246, was observed only in RT 078 probably due to the loss of the corresponding genes in other reference genomes. The evidence of production of high levels of both PPEP-1 substrates in this RT only supports the exclusive role of PPEP-1 in this representative and confirms the distinctive pathophysiological mechanisms from RTs 027 and 176.

l-Histidine biosynthesis is an ancient metabolic pathway present in bacteria, archaea, lower eukaryotes, and plants, and several proteins involved in this synthetic pathway were observed in this study. The pathway is regulated at the first committed step by hetero-oligomeric complex HisG/HisZ. HisG (C9YLE5) acting as aminoacyl-tRNA synthetase catalyzes the condensation of ATP and 5-phosphoribose 1-diphosphate to form N’-(5′-phosphoribosyl)-ATP (PR-ATP) and has a crucial role in the pathway because the rate of histidine biosynthesis seems to be controlled primarily by regulation of HisG enzymatic activity [41]. HisZ (C9YLE4) is an ATP phosphoribosyltransferase regulatory subunit essential for the catalytic activity of the whole complex [42]. In addition, the global repressor CodY, responsible also for suppressing of 19.6-kb Pathogenicity Locus, negatively regulates h*isZ* gene expression [43]. In this study, high levels of both of these proteins were determined in all representative strains possessing *tcdA* and *tcdB* genes. Moreover, in non-toxigenic representative strain the levels remained undetected, emphasizing the role of histidine biosynthesis in the virulence of *C. difficile*.

## Conclusions

Comparative proteomic analysis using label-free quantification (LFQ) of proteomes released from in vitro cultured *C. difficile* RTs 001, 005, 010, 012, 014, 027, 078, and 176 revealed several protein groups displaying varying protein levels between individual PCR ribotypes. These differences point to a new direction for studies aimed at the elucidation of the mechanisms underlining pathogenicity. The higher expression and/or unique expression of proteins linked to pathogenicity or iron metabolism support clinical importance and genetic relatedness of RTs 027 and 176 at the proteomic level. The nucleic acid zinc binding protein, cell wall binding repeat 2 family, Sigma-54 dependent regulatory protein and FliE were suggested as potential novel biomarkers of virulence based on differential expression among PCR ribotypes in this study.

## Additional file

**Additional file 1: **Detailed description of mass spectrometry procedures and proteomic results.

## Abbreviations

LFQ: label-free quantification
CDI: *Clostridium difficile* infection
DIGE: difference gel electrophoresis
TcdA: *Clostridium difficile* toxin A
TcdB: *Clostridium difficile* toxin B
MS: mass spectrometry
SNP: Single Nucleotide Polymorphism
GDH: glutamate dehydrogenase
MTZ: metronidazole
OD: optical density
CFU: colony-forming unit
FASP: filter aided sample preparation
TCEP: Tris (2-carboxyethyl) phosphine hydrochloride
ACN: acetonitrile
FA: formic acid
FDR: false discovery rate
CdtA: binary toxin A
CdtB: binary toxin B
PPEP-1: Pro–Pro endopeptidase 1
CdtR: orphan response regulator
NAP1/027: North American pulse-field type 1, PCR ribotype 027
RT: ribotype

## References

[1] Warny M, Pepin J, Fang A, Killgore G, Thompson A, Brazier J (2005). Toxin production by an emerging strain of *Clostridium difficile* associated with outbreaks of severe disease in North America and Europe. Lancet Lond Engl..

[2] Moura H, Terilli RR, Woolfitt AR, Williamson YM, Wagner G, Blake TA (2013). Proteomic analysis and label-free quantification of the large *Clostridium difficile* toxins. Int J Proteom..

[3] Stevenson E, Minton NP, Kuehne SA (2015). The role of flagella in *Clostridium difficile* pathogenicity. Trends Microbiol.

[4] Chilton CH, Gharbia SE, Fang M, Misra R, Poxton IR, Borriello SP (2014). Comparative proteomic analysis of *Clostridium difficile* isolates of varying virulence. J Med Microbiol.

[5] Wright A, Wait R, Begum S, Crossett B, Nagy J, Brown K (2005). Proteomic analysis of cell surface proteins from *Clostridium difficile*. Proteomics.

[6] Boetzkes A, Felkel KW, Zeiser J, Jochim N, Just I, Pich A (2012). Secretome analysis of Clostridium difficile strains. Arch Microbiol.

[7] Chen J-W, Scaria J, Mao C, Sobral B, Zhang S, Lawley T (2013). Proteomic comparison of historic and recently emerged hypervirulent *Clostridium difficile* strains. J Proteome Res.

[8] Megger DA, Bracht T, Meyer HE, Sitek B (2013). Label-free quantification in clinical proteomics. Biochim Biophys Acta BBA Proteins.

[9] Cox J, Hein MY, Luber CA, Paron I, Nagaraj N, Mann M (2014). Accurate proteome-wide label-free quantification by delayed normalization and maximal peptide ratio extraction, termed MaxLFQ. Mol Cell Proteom.

[10] Glatter T, Ahrné E, Schmidt A (2015). Comparison of different sample preparation protocols reveals lysis buffer-specific extraction biases in gram-negative bacteria and human cells. J Proteome Res.

[11] Little RH, Grenga L, Saalbach G, Howat AM, Pfeilmeier S, Trampari E (2016). Adaptive remodeling of the bacterial proteome by specific ribosomal modification regulates Pseudomonas infection and niche colonisation. PLoS Genet.

[12] Rosenberg A, Soufi B, Ravikumar V, Soares NC, Krug K, Smith Y, et al. Phosphoproteome dynamics mediate revival of bacterial spores. BMC Biol. 2015;13. http://www.biomedcentral.com/1741-7007/13/76. Accessed 5 Mar 2017. 10.1186/s12915-015-0184-7 PMC4574613 26381121

[13] Krutova M, Nyc O, Matejkova J, Allerberger F, Wilcox MH, Kuijper EJ. Molecular characterisation of Czech Clostridium difficile isolates collected in 2013–2015. Int J Med Microbiol. 2016. http://linkinghub.elsevier.com/retrieve/pii/S1438422116301266. Accessed 29 Mar 2016. 10.1016/j.ijmm.2016.07.003 27519407

[14] Rao K, Micic D, Natarajan M, Winters S, Kiel MJ, Walk ST (2015). *Clostridium difficile* ribotype 027: relationship to age, detectability of toxins a or b in stool with rapid testing, severe infection, and mortality. Clin Infect Dis.

[15] He M, Sebaihia M, Lawley TD, Stabler RA, Dawson LF, Martin MJ (2010). Evolutionary dynamics of *Clostridium difficile* over short and long time scales. Proc Natl Acad Sci.

[16] Davies KA, Ashwin H, Longshaw CM, Burns DA, Davis GL, Wilcox MH, et al. Diversity of *Clostridium difficile* PCR ribotypes in Europe: results from the European, multicentre, prospective, biannual, point-prevalence study of *Clostridium difficile* infection in hospitalised patients with diarrhoea (EUCLID), 2012 and 2013. Eurosurveillance. 2016;21. http://www.eurosurveillance.org/ViewArticle.aspx?ArticleId=22536. Accessed 9 Mar 2017. 10.2807/1560-7917.ES.2016.21.29.30294 27470194

[17] Stubbs SL, Brazier JS, O’Neill GL, Duerden BI (1999). PCR targeted to the 16S-23S rRNA gene intergenic spacer region of Clostridium difficile and construction of a library consisting of 116 different PCR ribotypes. J Clin Microbiol.

[18] Wiśniewski JR, Zougman A, Mann M (2009). Combination of FASP and StageTip-based fractionation allows in-depth analysis of the hippocampal membrane proteome. J Proteome Res.

[19] Smith PK, Krohn RI, Hermanson GT, Mallia AK, Gartner FH, Provenzano MD (1985). Measurement of protein using bicinchoninic acid. Anal Biochem.

[20] Cox J, Neuhauser N, Michalski A, Scheltema RA, Olsen JV, Mann M (2011). Andromeda: a peptide search engine integrated into the MaxQuant environment. J Proteome Res.

[21] Huang DW, Sherman BT, Lempicki RA (2009). Systematic and integrative analysis of large gene lists using DAVID bioinformatics resources. Nat Protoc.

[22] Petersen TN, Brunak S, von Heijne G, Nielsen H (2011). SignalP 4.0: discriminating signal peptides from transmembrane regions. Nat Methods.

[23] Bendtsen JD, Kiemer L, Fausbøll A, Brunak S (2005). Non-classical protein secretion in bacteria. BMC Microbiol.

[24] Kazmierczak MJ, Wiedmann M, Boor KJ (2005). Alternative sigma factors and their roles in bacterial virulence. Microbiol Mol Biol Rev.

[25] Stabler RA, He M, Dawson L, Martin M, Valiente E, Corton C (2009). Comparative genome and phenotypic analysis of Clostridium difficile 027 strains provides insight into the evolution of a hypervirulent bacterium. Genome Biol.

[26] Stabler RA, Gerding DN, Songer JG, Drudy D, Brazier JS, Trinh HT (2006). Comparative phylogenomics of Clostridium difficile reveals clade specificity and microevolution of hypervirulent strains. J Bacteriol.

[27] Valiente E, Dawson LF, Cairns MD, Stabler RA, Wren BW (2012). Emergence of new PCR ribotypes from the hypervirulent *Clostridium difficile* 027 lineage. J Med Microbiol.

[28] Lyon SA, Hutton ML, Rood JI, Cheung JK, Lyras D (2016). CdtR regulates TcdA and TcdB production in *Clostridium difficile*. PLOS Pathog..

[29] Skaar EP (2010). The battle for iron between bacterial pathogens and their vertebrate hosts. PLoS Pathog.

[30] Miethke M, Marahiel MA (2007). Siderophore-based iron acquisition and pathogen control. Microbiol Mol Biol Rev.

[31] Ho TD, Ellermeier CD (2015). Ferric uptake regulator fur control of putative iron acquisition systems in *Clostridium difficile*. J Bacteriol.

[32] Chong PM, Lynch T, McCorrister S, Kibsey P, Miller M, Gravel D (2014). Proteomic analysis of a NAP1 *Clostridium difficile* clinical isolate resistant to metronidazole. PLoS ONE.

[33] Minamino T, Yamaguchi S, Macnab RM (2000). Interaction between FliE and FlgB, a proximal rod component of the flagellar basal body of Salmonella. J Bacteriol.

[34] Twine SM, Reid CW, Aubry A, McMullin DR, Fulton KM, Austin J (2009). Motility and flagellar glycosylation in *Clostridium difficile*. J Bacteriol.

[35] Faulds-Pain A, Twine SM, Vinogradov E, Strong PCR, Dell A, Buckley AM (2014). The post-translational modification of the *Clostridium difficile* flagellin affects motility, cell surface properties and virulence. Mol Microbiol.

[36] Tasteyre A, Karjalainen T, Avesani V, Delmée M, Collignon A, Bourlioux P (2000). Phenotypic and genotypic diversity of the flagellin gene (fliC) among *Clostridium difficile* isolates from different serogroups. J Clin Microbiol.

[37] Tasteyre A, Karjalainen T, Avesani V, Delmée M, Collignon A, Bourlioux P (2001). Molecular characterization of fliD gene encoding flagellar cap and its expression among *Clostridium difficile* isolates from different serogroups. J Clin Microbiol.

[38] Hensbergen PJ, Klychnikov OI, Bakker D, van Winden VJC, Ras N, Kemp AC (2014). A novel secreted metalloprotease (CD2830) from *Clostridium difficile* cleaves specific proline sequences in LPXTG cell surface proteins. Mol Cell Proteom.

[39] Hensbergen PJ, Klychnikov OI, Bakker D, Dragan I, Kelly ML, Minton NP (2015). Clostridium difficile secreted Pro-Pro endopeptidase PPEP-1 (ZMP1/CD2830) modulates adhesion through cleavage of the collagen binding protein CD2831. FEBS Lett.

[40] Peltier J, Shaw HA, Couchman EC, Dawson LF, Yu L, Choudhary JS (2015). Cyclic diGMP regulates production of sortase substrates of *Clostridium difficile* and their surface exposure through ZmpI protease-mediated cleavage. J Biol Chem.

[41] Delorme C, Ehrlich SD, Renault P (1992). Histidine biosynthesis genes in *Lactococcus lactis* subsp. lactis. J Bacteriol.

[42] Sissler M, Delorme C, Bond J, Ehrlich SD, Renault P, Francklyn C (1999). An aminoacyl-tRNA synthetase paralog with a catalytic role in histidine biosynthesis. Proc Natl Acad Sci USA.

[43] Dineen SS, McBride SM, Sonenshein AL (2010). Integration of metabolism and virulence by *Clostridium difficile* CodY. J Bacteriol.

