# Supplementary material for: Analysis of proteomes released from in vitro cultured eight Clostridium difficile PCR ribotypes revealed specific expression in PCR ribotypes 027 and 176 confirming their genetic relatedness and clinical importance at the proteomic level
Source: Gut Pathog. 2017 Aug 14;9:45. doi: 10.1186/s13099-017-0194-9 (PMC5556371; doi:10.1186/s13099-017-0194-9)
Supplement: Supplementary file 1 — Additional file 1: Detailed description of mass spectrometry procedures and proteomic results. [file 13099_2017_194_MOESM1_ESM.doc]

Additional file 1.

# Analysis of proteomes released from *in vitro* cultured eight *Clostridium difficile* PCR ribotypes revealed specific expression in PCR ribotypes 027 and 176 confirming their genetic relatedness and clinical importance at the proteomic level.

**Jiri Dresler^*^, Marcela Krutova, Alena Fucikova, Jana Klimentova, Veronika Hruzova, Miloslava Duracova, Katerina Houdkova, Barbora Salovska, Jana Matejkova, Martin Hubalek, Petr Pajer, Libor Pisa and Otakar Nyc**

*** Correspondence:** Jiri Dresler: jiri.dresler@gmail.com

## S 1.1 LC-MS/MS analysis

An UltiMate 3000 RSLCnano system controlled by Chromeleon software (Dionex, USA) was used for chromatography separation. 1 µL of each sample (10x diluted) was loaded onto a PepMap100 C18, 3 µm, 100 Å, 0.075 × 20 mm trap column (Dionex) at 5 µL/min for 5 min. Peptides were separated on a PepMap RSLC C18, 2 µm, 100 Å, 0.075 × 150 mm analytical column (Dionex) by a gradient formed by mobile phase A (0.1% formic acid, FA) and mobile phase B (80% ACN, 0.1% FA), running from 4 to 34% in 68 min, and from 34 to 55% of mobile phase B in 21 min at a flow rate of 0.3 µL/min at 40 °C. Eluted peptides were on-line electrosprayed into Q-Exactive mass spectrometer using a Nanospray Flex ion source (Thermo Scientific, Bremen, Germany). Positive ion full scan MS spectra (*m/z* 350-1650) were acquired using a 1×10^6^ AGC target in the Orbitrap at 70 000 resolution. Top 12 precursors of charge state ≥2 and threshold intensity of 5×10^4^ counts were selected for HCD fragmentation, with a dynamic exclusion window of 30 s. The isolation window of 1.6 Da and normalized CE of 27 was used. Each MS/MS spectrum was acquired at resolution of 17,500, with a 10^5^ AGC target and a maximum 100 ms injection time.

HCD MS/MS spectra were searched in Proteome Discoverer software v. 1.4 (Thermo Scientific) using MASCOT (Matrix Science, London, UK). The reference proteome set of *Peptoclostridium difficile* strain R20291 was downloaded from UniProt/KB in July 2015 and merged with common contaminants file downloaded from the MaxQuant web page (<http://www.maxquant.org/downloads.htm>); the merged database contained 3754 sequences. The tryptic specificity was set with maximum of 2 missed cleavages. The mass tolerance was set to 10 ppm for precursors and 20 mmu for product ions. Cys carbamidomethylation was set as a fixed modification and methionin oxidation was set as variable modification. Spectra explained with a cut-off score 0.95 were extracted by Skyline software from the MASCOT .dat file and stored in an MS/MS library.

## S 1.2 Targeted mass spectrometry analysis of TcdA and TcdB

Based on the analyzed tryptic digests of TcdA and TcdB, the candidate peptides were compared with the whole UniProt database (ver. 20140907) using the homemade script running in BioPearl to ensure their specificity. Finally, four tryptic peptides (two for each protein) were considered as signature for TcdA and TcdB protein and purchased as isotopically labeled synthetic equivalents (SpikeTides TQL, JPT Peptide Technologies, Berlin, Germany): NIAYNYTDESNNK+Label:13C(6)15N(2)(13) and NNFYFDANNESK+Label:13C(6)15N(2)(12) for TcdA and TPYDSVLFQK+Label:13C(6)15N(2)(10) and NNNLTPVEK+Label:13C(6)15N(2)(9) for TcdB.

These peptides were subsequently used in known concentrations as spiked standards either for background interferences measurement or for the absolute quantification of these peptides across the individual samples. The synthetic signature peptides were spiked into the pooled sample from toxin negative strains (background sample) in known concentration ranging from 40 to 160 fmol/µl per one injection. Three replicates for each concentration step were measured in the order from the low to the highest concentration. The coefficient of variation of these three replicates was always below 20 percent. The linear regression analysis on the observed native:labeled peak area ratios versus concentration was performed to prepare the curve for testing of background versus peptide interactions (see Additional file 1: Table S2 and Figure S1). Finally, the concentration of 40 fmol of heavy labeled peptides were spiked into each sample and ratio of intensities between analyzed sample and toxin negative control (samples from ribotype 010) was counted, for the intensities values see Additional file 1: Table S3.

## S 1.3 LFQ - Raw data processing of *C. difficile* proteins

Raw files acquired in S 1.1 were further analyzed in MaxQuant [1] (version 1.5.3.30) and the Andromeda was used as search engine [2] to search the detected features against the *C. difficile* strain R20291 (PCR-ribotype 027) or against *C. difficile* strain 630 (PCR-ribotype 012) databases downloaded from Uniprot ( June 17^th^ 2015). Only tryptic peptides that were at least seven amino acids in length with up to two missed cleavages were considered. The initial allowed mass tolerance was set to 4.5 ppm at the MS level and 0.5 Da at the MS/MS level. The oxidation of methionine was set as variable modification and carbamidomethylation of cysteine was set as a fixed modification. A false discovery rate (FDR) of 1 % was imposed for peptide-spectrum matches (PSMs) and protein identification using a target–decoy approach. Relative quantification was performed using the default parameters of the MaxLFQ algorithm [3] with the minimum ratio count set to 2.

## S 1.4 LFQ Data analysis of *C. difficile* proteins

The “proteinGroups.txt” MaxQuant output file was uploaded into Perseus [3] (version 1.5.2.6) for further filtering and statistical analysis. Decoy hits, proteins only identified by site, and potential contaminants were removed. Only those protein groups quantified in at least two replicates of at least one ribotype representative triplicate were considered for further log_2_ transformation of LFQ intensities.

| Sample number | Ribotype | OD at the beginning of the cultivation | | OD at the end of the cultivation | Protein quantity (µg) |
| --- | --- | --- | --- | --- | --- |
| 1 | 027 |  | 1.99 | 19.33 | 612 |
| 2 | 010 |  | 1.99 | 19.67 | 727 |
| 3 | 014 |  | 1.99 | 22.33 | 575 |
| 4 | 078 |  | 1.99 | 31.83 | 688 |
| 5 | 012 |  | 1.99 | 22.00 | 675 |
| 6 | 005 |  | 1.99 | 22.17 | 613 |
| 7 | 176 |  | 1.99 | 14.83 | 693 |
| 8 | 001 |  | 1.99 | 21.67 | 767 |
| 9 | 027 |  | 1.99 | 16.83 | 704 |
| 10 | 010 |  | 1.99 | 21.67 | 1058 |
| 11 | 014 |  | 1.99 | 21.33 | 756 |
| 12 | 078 |  | 1.99 | 30.17 | 777 |
| 13 | 012 |  | 1.99 | 19.33 | 831 |
| 14 | 005 |  | 1.99 | 14.33 | 800 |
| 15 | 176 |  | 1.99 | 13.83 | 585 |
| 16 | 001 |  | 1.99 | 23.17 | 851 |
| 17 | 027 |  | 1.99 | 18.67 | 663 |
| 18 | 010 |  | 1.99 | 23.83 | 611 |
| 19 | 014 |  | 1.99 | 20.17 | 722 |
| 20 | 078 |  | 1.99 | 22.50 | 903 |
| 21 | 012 |  | 1.99 | 16.67 | 745 |
| 22 | 005 |  | 1.99 | 19.67 | 906 |
| 23 | 176 |  | 1.99 | 13.33 | 447 |
| 24 | 001 |  | 1.99 | 21.67 | 743 |

Table S1: List of samples with the numbers of OD values at the beginning and at the end of the cultivation with their protein yields

| peptide | Signal intensities | | | | |
| --- | --- | --- | --- | --- | --- |
|  | background sample | | 40fmol | 80fmol | 160fmol |
| NIAYNYTDESNNK | 2363110 | 2153524 | | 5282690 | 10033673 |
| NNFYFDANNESK | 1895676 | 4061295 | | 7207577 | 18919467 |
| TPYDSVLFQK | 1522596 | 18967570 | | 40991403 | 86171121 |
| NNNLTPVEK | 1346579 | 1842809 | | 3618921 | 6420758 |

Table S2: Peptide intensities of TcdA and TcdB synthetic peptides spiked into sample of the toxin negative strain (background sample)

|  |  |  | **LFQ intensities (medians)** | | | | | | | |  |
| --- | --- | --- | --- | --- | --- | --- | --- | --- | --- | --- | --- |
|  | **Protein IDs** |  | **PCR ribotype 010** | **PCR ribotype 014** | **PCR ribotype 012** | **PCR ribotype 005** | **PCR ribotype 001** | **PCR ribotype 078** | **PCR ribotype 027** | **PCR ribotype 176** |  |
|  | C9YHF8 |  | 23.303267 | 23.726816 | 23.536707 | 23.941275 | 23.359711 | 24.521511 | 23.259830 | 23.267710 |  |
|  | C9YHG2 |  | 22.386663 | 22.666939 | 23.929992 | 22.256926 | NaN | 23.484726 | 22.745605 | 22.470702 |  |
|  | C9YHH7 |  | 23.359170 | 23.781368 | 23.630009 | 24.227659 | 23.815092 | 22.860678 | 23.961905 | 23.891432 |  |
|  | C9YHJ6 |  | 25.165035 | 26.086143 | 25.705881 | 25.290668 | 25.268887 | 26.832676 | 25.459984 | 25.808086 |  |
|  | C9YHJ8 |  | 23.716805 | 24.233009 | 24.815580 | 25.575050 | 23.968424 | 25.211821 | 25.111238 | 24.684906 |  |
|  | C9YHK0 |  | 22.976768 | 23.683460 | 23.874834 | 23.233377 | NaN | 23.099665 | 22.822025 | 24.717537 |  |
|  | C9YHK6 |  | 26.524384 | 27.011160 | 26.673441 | 26.439062 | 26.710039 | 25.157885 | NaN | NaN |  |
|  | C9YHL0 |  | 25.572508 | 26.025953 | 26.005196 | 25.682924 | 26.080421 | 26.012695 | 25.949846 | 25.991444 |  |
|  | C9YHL1 |  | 23.069220 | 22.790226 | 22.545477 | 23.354408 | NaN | NaN | 23.404575 | 23.258106 |  |
|  | C9YHL4 |  | 28.721134 | 27.879469 | 28.686056 | 29.181345 | 29.168205 | 27.632563 | 28.444468 | 28.601624 |  |
|  | C9YHL5 |  | 28.775333 | 27.640377 | 29.894638 | 30.267294 | 28.540194 | 28.462013 | 28.402960 | 28.524736 |  |
|  | C9YHL6 |  | 29.721216 | 28.883204 | 29.965679 | 30.196480 | 29.676184 | 28.559359 | 29.477633 | 29.555542 |  |
|  | C9YHL7 |  | 31.608362 | 31.873240 | 31.805128 | 31.902933 | 31.857754 | 32.355553 | 31.569099 | 31.578892 |  |
|  | C9YHL8 |  | 23.709042 | 27.812866 | 22.293421 | 23.905640 | 22.133171 | 26.129002 | 22.969391 | 22.489885 |  |
|  | C9YHL9 |  | 24.880590 | 24.337152 | 25.919436 | 25.138369 | 24.595312 | 24.242054 | 25.014696 | 24.808920 |  |
|  | C9YHM2 |  | 25.161924 | 24.615602 | 24.855240 | 25.404284 | 25.331013 | 25.705935 | 25.510084 | 25.545689 |  |
|  | C9YHM3 |  | 25.646940 | 24.964109 | 26.029453 | 24.971058 | 25.377527 | 24.787458 | 25.197344 | 25.783417 |  |
|  | C9YHM5 |  | 26.225086 | 25.403698 | 25.005003 | 26.441597 | 25.784491 | 26.004017 | 26.499266 | 26.069727 |  |
|  | C9YHM6 |  | 24.861502 | 24.551510 | 26.569860 | 25.738770 | 24.718897 | 25.811348 | 25.030401 | 25.065502 |  |
|  | C9YHM7 |  | 23.686777 | 23.447710 | 26.131456 | 23.663511 | 23.524324 | 23.570743 | 23.678419 | 23.859380 |  |
|  | C9YHM8 |  | 25.025043 | 24.490816 | 25.750368 | 24.418283 | 25.067924 | 27.345028 | 24.847614 | 24.217194 |  |
|  | C9YHM9 |  | 26.521662 | 26.172245 | 27.786188 | 26.790792 | 26.316517 | 27.454504 | 26.526148 | 26.506805 |  |
|  | C9YHN0 |  | 23.709566 | 23.677128 | 22.899937 | 23.435820 | 23.716487 | NaN | 22.795658 | 23.559438 |  |
|  | C9YHN1 |  | 23.343672 | 24.385942 | 25.148140 | 23.250652 | 22.673725 | 22.611668 | 23.384954 | 22.855953 |  |
|  | C9YHN2 |  | 24.624107 | 24.396107 | 24.840525 | 25.004017 | 24.756897 | 24.590809 | 24.686777 | 24.897583 |  |
|  | C9YHN4 |  | 24.682635 | 23.797586 | 25.779314 | 26.181051 | 24.728270 | 27.977819 | 25.044144 | 24.506578 |  |
|  | C9YHN6 |  | 24.867990 | 24.454000 | 25.994835 | 25.154064 | 24.795755 | 24.492518 | 24.971102 | 24.817484 |  |
|  | C9YHN7 |  | 26.042830 | 25.625502 | 26.022802 | 25.646584 | 25.493841 | 26.490402 | 25.621483 | 25.740675 |  |
|  | C9YHN8 |  | 26.562862 | 26.621567 | 27.617159 | 26.475544 | 26.955267 | 26.611893 | 26.836655 | 26.590065 |  |
|  | C9YHP0 |  | 27.545248 | 27.849596 | 27.979673 | 28.325174 | 27.989506 | 28.555998 | 27.846491 | 27.844877 |  |
|  | C9YHP1 |  | 22.753748 | 23.078419 | 24.648724 | 22.406031 | 23.080763 | 22.300719 | 22.759388 | 22.722281 |  |
|  | C9YHP2 |  | 24.060984 | 23.499113 | 26.472990 | 24.139305 | 24.039757 | 24.132496 | 24.148954 | 24.035822 |  |
|  | C9YHP3 |  | 26.260351 | 25.845152 | 25.410604 | 26.817875 | 25.688671 | 26.563429 | 26.004961 | 26.262323 |  |
|  | C9YHP4 |  | 23.712404 | 24.482470 | 24.962523 | 24.136414 | 24.250391 | 25.889622 | 24.162464 | 23.911411 |  |
|  | C9YHP5 |  | 23.837881 | 23.237546 | 25.442909 | 23.242897 | 23.865896 | 24.559292 | 24.169668 | 24.114420 |  |
|  | C9YHP7 |  | 30.362648 | 29.481930 | 30.185181 | 29.924932 | 29.849403 | 28.871441 | 29.493620 | 30.081570 |  |
|  | C9YHQ0 |  | 26.338055 | 26.837738 | 26.320860 | 26.581327 | 26.598467 | 26.558664 | 26.157095 | 26.265261 |  |
|  | C9YHQ1 |  | 22.363031 | 21.774105 | 22.306831 | 22.277107 | 22.124773 | 22.631321 | NaN | 22.391735 |  |
|  | C9YHQ2 |  | 26.619610 | 26.477985 | 26.090248 | 26.790047 | 26.739182 | 28.083803 | 26.760784 | 26.721819 |  |
|  | C9YHQ4 |  | 24.775251 | 24.683889 | 24.062582 | 24.601501 | 24.648285 | 24.553268 | 25.017557 | 24.734486 |  |
|  | C9YHQ5 |  | 25.818558 | 25.603569 | 26.113308 | 26.006096 | 24.959297 | 25.085577 | 25.233706 | 25.619888 |  |
|  | C9YHR2 |  | 25.130730 | 24.353128 | 24.135239 | 25.694290 | 25.094423 | 25.297087 | 25.337288 | 25.387356 |  |
|  | C9YHR4 |  | 26.230978 | 27.465755 | 26.285686 | 25.659407 | 25.826338 | 25.554792 | 26.209831 | 26.609781 |  |
|  | C9YHS0 |  | 26.159328 | 26.070322 | 26.232754 | 25.683783 | 25.212450 | 25.660223 | 25.640308 | 26.363258 |  |
|  | C9YHS3 |  | 30.706850 | 29.581902 | 29.385210 | 29.328751 | 30.497528 | 30.035511 | 29.560579 | 29.663498 |  |
|  | C9YHS4 |  | 28.180161 | 26.791662 | 25.665600 | 26.765596 | 27.719275 | 27.099188 | 27.099689 | 27.509602 |  |
|  | C9YHS5 |  | 27.199947 | 25.936182 | 24.328688 | 25.857782 | 26.981853 | 25.973030 | 26.181412 | 26.625195 |  |
|  | C9YHS6 |  | 29.475025 | 28.391493 | 27.659557 | 28.083397 | 29.163534 | 28.620203 | 28.466883 | 28.917269 |  |
|  | C9YHS7 |  | 23.076611 | NaN | 23.326868 | NaN | NaN | 25.042475 | NaN | NaN |  |
|  | C9YHS8 |  | NaN | 24.449092 | 24.493689 | 23.880031 | 23.937202 | 24.293898 | 23.763587 | 24.137430 |  |
|  | C9YHT1 |  | 28.613123 | 29.160700 | 28.868353 | 28.385826 | 28.677916 | 28.761513 | 28.818394 | 28.813019 |  |
|  | C9YHT6 |  | 23.765673 | 25.255894 | 25.264133 | 24.217562 | 23.844219 | 23.744169 | 24.135475 | 23.740675 |  |
|  | C9YHT8 |  | 28.402594 | 27.506428 | 27.469486 | 28.020197 | 27.836414 | 26.721819 | 28.116444 | 27.683580 |  |
|  | C9YHV4 |  | 23.983806 | 24.283056 | 24.193652 | 24.264349 | 24.539610 | 24.103176 | 24.394733 | 24.581989 |  |
|  | C9YHY0 |  | 26.535980 | 28.016962 | 27.158463 | 26.982180 | 26.522635 | 25.632952 | 26.920139 | 27.430218 |  |
|  | C9YHY4 |  | 25.927822 | 28.581758 | 26.599319 | 26.591064 | 25.633394 | 26.367481 | 26.969280 | 27.294039 |  |
|  | C9YHY7 |  | 27.313631 | 23.371723 | 26.366331 | 26.610344 | 26.372671 | 25.955290 | 25.677692 | 26.134182 |  |
|  | C9YHY9 |  | 36.045452 | 34.598789 | 35.869541 | 35.407841 | 36.163651 | 36.821571 | 35.641865 | 35.233921 |  |
|  | C9YHZ2 |  | 29.513165 | 29.275177 | 28.492359 | 28.525370 | 28.475800 | 29.837301 | 28.824263 | 29.040014 |  |
|  | C9YHZ3 |  | 27.644508 | 26.201313 | 27.047289 | 26.706356 | 26.917980 | 24.327750 | 26.156574 | 26.439507 |  |
|  | C9YHZ4 |  | 23.174719 | 23.872808 | 21.880207 | 24.703297 | 24.040762 | 24.760607 | 24.000875 | 23.827424 |  |
|  | C9YHZ5 |  | NaN | NaN | NaN | 22.806425 | 23.085617 | 23.649656 | 23.083038 | NaN |  |
|  | C9YHZ6 |  | 28.516718 | 28.223291 | 28.348745 | 28.711681 | 28.323500 | 28.664516 | 28.008171 | 28.030548 |  |
|  | C9YI02 |  | 29.922098 | 30.798307 | 30.086767 | 29.718901 | 29.375589 | 30.969595 | 29.417236 | 29.811428 |  |
|  | C9YI03 |  | 32.620693 | 32.989967 | 33.364761 | 32.912296 | 32.351425 | 34.010971 | 31.986309 | 32.202011 |  |
|  | C9YI05 |  | 24.228025 | 23.749397 | 24.773542 | 23.419827 | 23.314651 | 22.912580 | 23.494604 | 24.131556 |  |
|  | C9YI17 |  | NaN | NaN | 22.481522 | NaN | NaN | 27.796682 | NaN | NaN |  |
|  | C9YI24 |  | 25.220886 | 26.396122 | 26.592491 | 26.717119 | 25.364027 | 25.222214 | 25.245213 | 26.216324 |  |
|  | C9YI25 |  | 27.356239 | 28.012066 | 26.885765 | 28.299900 | 27.310427 | 29.798941 | 27.315100 | 27.082434 |  |
|  | C9YI29 |  | 22.839209 | 22.797334 | NaN | 23.447332 | NaN | 23.388566 | NaN | 23.244568 |  |
|  | C9YI31 |  | NaN | NaN | NaN | NaN | NaN | NaN | NaN | 23.959253 |  |
|  | C9YI32 |  | NaN | 23.901686 | NaN | 24.803314 | NaN | 23.736860 | NaN | NaN |  |
|  | C9YI34 |  | NaN | 23.520254 | NaN | 23.585661 | NaN | NaN | 25.456791 | 25.718113 |  |
|  | C9YI37 |  | NaN | 22.797052 | NaN | 23.025839 | NaN | NaN | 24.959431 | 24.996494 |  |
|  | C9YI39 |  | NaN | 25.829874 | NaN | 27.111477 | NaN | 20.800808 | 27.408922 | 27.857687 |  |
|  | C9YI40 |  | NaN | 26.930103 | NaN | 28.237175 | NaN | NaN | 28.874376 | 29.272642 |  |
|  | C9YI44 |  | NaN | NaN | NaN | 23.868428 | NaN | NaN | 23.784966 | 24.160348 |  |
|  | C9YI45 |  | NaN | 28.582441 | NaN | 29.597118 | NaN | 24.478895 | 30.848343 | 31.331808 |  |
|  | C9YI47 |  | 22.697239 | 31.367603 | 24.821966 | 32.072048 | 23.016245 | 24.451488 | 33.497265 | 33.418655 |  |
|  | C9YI56 |  | NaN | 23.397543 | NaN | 21.615444 | NaN | NaN | 23.329372 | 24.206055 |  |
|  | C9YI57 |  | NaN | NaN | NaN | NaN | NaN | NaN | 24.726557 | 25.183704 |  |
|  | C9YI63 |  | NaN | 24.351984 | NaN | 24.203741 | NaN | NaN | 25.450701 | 25.948732 |  |
|  | C9YI65 |  | NaN | 26.915018 | NaN | 27.881163 | NaN | NaN | 28.366571 | 28.863310 |  |
|  | C9YI69 |  | NaN | NaN | NaN | 24.374578 | NaN | NaN | 24.180330 | 24.368664 |  |
|  | C9YI79 |  | NaN | 26.010041 | NaN | 26.517553 | NaN | NaN | 27.145304 | 27.733438 |  |
|  | C9YI80 |  | NaN | 25.728788 | NaN | 25.692215 | NaN | NaN | 27.176075 | 27.568483 |  |
|  | C9YI84 |  | 24.834366 | 24.290316 | 24.979282 | 25.803782 | 25.196518 | 25.980415 | 25.233961 | 25.119297 |  |
|  | C9YI85 |  | 24.290668 | NaN | 23.680674 | 23.171150 | 23.469653 | NaN | 22.679922 | 22.551361 |  |
|  | C9YI90 |  | 28.974787 | 27.235167 | 25.603569 | 26.875725 | 27.590994 | 29.826159 | 27.870384 | 27.831348 |  |
|  | C9YIA5 |  | 27.200041 | 24.704931 | 27.470417 | 26.570539 | 26.650888 | 23.906823 | 27.280119 | 27.193382 |  |
|  | C9YIB0 |  | 22.040127 | 22.399057 | 21.846018 | 21.656322 | 22.626450 | 23.614029 | 22.188207 | 21.560092 |  |
|  | C9YIB4 |  | 24.132105 | NaN | 22.695820 | 23.024914 | 22.853329 | 24.401976 | 22.695160 | 22.401585 |  |
|  | C9YIE0 |  | 23.678453 | NaN | 23.595255 | NaN | NaN | NaN | NaN | NaN |  |
|  | C9YIE1 |  | 28.572067 | 28.202003 | 28.508015 | 28.210993 | 28.291407 | 28.264500 | 28.719013 | 28.572933 |  |
|  | C9YIE4 |  | 29.537512 | 24.228907 | 29.824430 | 19.697495 | 19.475770 | 28.079485 | 24.575829 | 26.447584 |  |
|  | C9YIG9 |  | 24.871162 | 24.357767 | 24.067268 | 24.346097 | 24.245903 | NaN | NaN | 23.220704 |  |
|  | C9YIH1 |  | 28.058273 | 26.271116 | 28.032757 | 27.380470 | 28.264053 | 27.360264 | 27.457872 | 27.747849 |  |
|  | C9YIH2 |  | 32.934753 | 31.644697 | 32.182842 | 32.297699 | 32.679802 | 33.096539 | 32.500996 | 32.620693 |  |
|  | C9YIH3 |  | 22.344160 | NaN | 22.526981 | 22.804089 | 22.703939 | NaN | 23.112686 | 23.323061 |  |
|  | C9YIH4 |  | 31.022938 | 30.833265 | 31.263941 | 31.045441 | 30.615089 | 31.280241 | 30.445988 | 31.400600 |  |
|  | C9YIH5 |  | 32.357677 | 31.341013 | 32.020554 | 31.786203 | 31.959406 | 31.274754 | 32.067566 | 32.281872 |  |
|  | C9YIH6 |  | 30.584951 | 28.681372 | 30.471600 | 29.909849 | 30.592812 | 29.547455 | 29.990913 | 31.276587 |  |
|  | C9YIH7 |  | 29.785875 | 28.139753 | 28.918747 | 28.747463 | 29.904406 | 29.432093 | 29.386936 | 29.896935 |  |
|  | C9YIH8 |  | 29.814655 | 27.915018 | 28.743778 | 28.760784 | 30.345831 | 29.264769 | 29.427343 | 29.889513 |  |
|  | C9YII0 |  | 33.710159 | 33.557049 | 33.329376 | 33.737816 | 34.298573 | 34.241116 | 33.644524 | 33.796917 |  |
|  | C9YII1 |  | 25.733994 | 26.164862 | 26.311121 | 26.131731 | 26.183439 | NaN | 26.455492 | 26.470541 |  |
|  | C9YII4 |  | 25.376434 | 25.702873 | 25.899521 | 25.442562 | 25.167372 | 26.026016 | 25.708488 | 25.874741 |  |
|  | C9YII7 |  | NaN | 24.165764 | 26.008875 | 27.863192 | NaN | NaN | 27.826750 | 27.397787 |  |
|  | C9YIP6 |  | 31.898975 | 23.114927 | 31.126448 | 30.559284 | 30.965611 | 29.308996 | 31.763786 | 30.743183 |  |
|  | C9YIQ6 |  | NaN | NaN | NaN | NaN | NaN | NaN | 23.108593 | 23.903986 |  |
|  | C9YIQ9 |  | NaN | 23.139431 | 23.509783 | 22.722527 | 22.654644 | NaN | 23.154812 | 23.937239 |  |
|  | C9YIS5 |  | NaN | NaN | 22.216467 | 23.683033 | 22.168388 | 24.614311 | 22.686888 | 22.575038 |  |
|  | C9YIS6 |  | 25.455538 | 26.050758 | 25.252848 | 24.647022 | 25.396955 | 25.767670 | 25.263382 | 25.293196 |  |
|  | C9YIT0 |  | 25.703032 | 27.451990 | 25.697899 | 26.631315 | 26.352068 | 28.385950 | 27.407545 | 27.520134 |  |
|  | C9YIT2 |  | NaN | NaN | 24.056559 | 25.459452 | NaN | NaN | NaN | NaN |  |
|  | C9YIT5 |  | 24.349083 | 24.053460 | 24.127388 | 25.017460 | 24.197905 | 24.419762 | 24.837255 | 24.629509 |  |
|  | C9YIU1 |  | NaN | 24.137039 | NaN | NaN | NaN | NaN | NaN | NaN |  |
|  | C9YIU2 |  | 24.549572 | NaN | 23.908115 | 24.087318 | 23.327591 | 24.476734 | 23.252747 | 22.933840 |  |
|  | C9YIU8 |  | 26.362272 | 27.829596 | 26.686457 | 27.188580 | 26.664787 | 27.524773 | 26.540466 | 26.743553 |  |
|  | C9YIU9 |  | 22.802485 | 24.350569 | 22.372590 | NaN | 22.799208 | NaN | 23.479141 | 23.587606 |  |
|  | C9YIV4 |  | NaN | 22.201469 | 22.449536 | 21.729702 | 21.762139 | 22.000687 | NaN | 22.106901 |  |
|  | C9YIW2 |  | NaN | 26.071182 | NaN | NaN | NaN | NaN | 21.982628 | 21.928066 |  |
|  | C9YIW6 |  | 27.880928 | 24.357296 | 29.900667 | 27.794455 | 28.865967 | 27.357414 | 29.834200 | 29.946983 |  |
|  | C9YIW7 |  | 26.952715 | 24.662371 | 28.367281 | 27.140535 | 27.861240 | NaN | 29.855030 | 29.533655 |  |
|  | C9YIX9 |  | 25.341503 | 23.981314 | 25.742012 | 25.198280 | 25.496374 | NaN | 25.136610 | 24.939077 |  |
|  | C9YJ35 |  | NaN | NaN | NaN | NaN | NaN | 25.519833 | 28.961601 | 29.516888 |  |
|  | C9YJ37 |  | NaN | NaN | 23.940647 | 21.039257 | NaN | 22.698269 | 29.699207 | 30.837933 |  |
|  | C9YJ63 |  | 26.179325 | 26.730396 | 26.472355 | 25.979609 | 26.065481 | 26.254074 | 25.616499 | 26.341129 |  |
|  | C9YJ65 |  | 25.755903 | 25.496677 | NaN | NaN | NaN | 24.800997 | 25.111597 | 25.522709 |  |
|  | C9YJ77 |  | 23.129213 | 22.585293 | 22.949383 | 22.687304 | 22.734238 | NaN | 22.550154 | 22.934469 |  |
|  | C9YJ79 |  | 24.617596 | 24.669743 | 24.947485 | 24.726088 | 24.433708 | 24.832001 | 24.221920 | 24.581530 |  |
|  | C9YJ83 |  | 24.714293 | 22.803724 | 23.977274 | 25.143709 | 24.421047 | 24.354744 | 23.566278 | 23.143242 |  |
|  | C9YJ84 |  | 24.598211 | 24.191368 | 24.004414 | 23.447332 | 24.708357 | NaN | 24.157614 | 23.910587 |  |
|  | C9YJ85 |  | 22.769129 | NaN | 22.393805 | 22.758717 | NaN | NaN | 24.175390 | 24.128490 |  |
|  | C9YJ86 |  | 34.390617 | 33.777843 | 34.164589 | 34.285610 | 34.536991 | 34.404133 | 33.925304 | 33.952202 |  |
|  | C9YJ93 |  | 23.749498 | 24.268852 | 25.032799 | 25.518454 | 23.970444 | 26.583481 | 23.938854 | 24.575483 |  |
|  | C9YJ96 |  | 32.491138 | 29.754204 | 32.225529 | 31.916698 | 32.469368 | 30.510036 | 31.799850 | 31.812912 |  |
|  | C9YJ97 |  | 29.192841 | 27.277462 | 29.517450 | 29.383995 | 29.457088 | 29.663736 | 29.137680 | 28.600170 |  |
|  | C9YJ98 |  | 24.060934 | NaN | 23.940020 | 23.535284 | 23.809999 | NaN | 24.035654 | 24.447521 |  |
|  | C9YJ99 |  | 24.537418 | NaN | 26.379230 | 26.690857 | 25.736086 | 25.270706 | 25.753916 | 24.963272 |  |
|  | C9YJA0 |  | 25.322063 | NaN | 26.698030 | 27.157787 | 26.356054 | 25.543509 | 26.101414 | 25.369795 |  |
|  | C9YJA1 |  | 26.609922 | 24.052998 | 27.538752 | 27.213915 | 26.896776 | 25.979107 | 26.505959 | 26.640583 |  |
|  | C9YJA3 |  | 24.621874 | NaN | 25.141138 | 24.052502 | 23.431036 | 22.730005 | 24.222952 | 24.731796 |  |
|  | C9YJA4 |  | 25.535313 | 23.580954 | 25.797289 | 25.477383 | 24.724371 | 24.714134 | 25.263060 | 25.547192 |  |
|  | C9YJA6 |  | NaN | NaN | 21.266594 | 21.054711 | NaN | 20.994058 | 21.218864 | 21.835382 |  |
|  | C9YJA7 |  | 27.527237 | 26.009560 | 27.934834 | 27.419086 | 26.759769 | 27.367447 | 26.554850 | 26.584055 |  |
|  | C9YJB2 |  | 27.236992 | 25.207283 | 27.515404 | 26.222086 | 27.692654 | 28.388456 | 26.983810 | 27.342741 |  |
|  | C9YJB3 |  | 25.721323 | 25.644329 | 25.460421 | 25.457106 | 25.632313 | 25.393818 | 25.583338 | 25.605709 |  |
|  | C9YJB4 |  | 25.076471 | 24.725204 | 25.082535 | 24.945076 | 25.522949 | 25.733912 | 24.993734 | 24.462202 |  |
|  | C9YJB7 |  | 23.657741 | 24.764004 | 23.744991 | 24.146936 | 23.634529 | NaN | 24.956509 | 23.818899 |  |
|  | C9YJC5 |  | 23.084255 | 23.625213 | 23.825319 | 23.581530 | 22.856504 | 23.504524 | 23.084936 | 23.050632 |  |
|  | C9YJC8 |  | 26.895506 | 27.463028 | 26.748425 | 27.521931 | 27.226740 | 26.905640 | 27.340874 | 27.098484 |  |
|  | C9YJD4 |  | 28.566162 | 28.151802 | 28.098936 | 27.712141 | 28.774736 | 28.008224 | 28.883612 | 28.238907 |  |
|  | C9YJD6 |  | 30.128294 | 29.837769 | 28.083143 | 25.790644 | 28.568701 | 29.015873 | 26.766356 | 27.807716 |  |
|  | C9YJD7 |  | 26.290405 | 27.256559 | 26.383554 | 25.146431 | 26.359844 | 26.478186 | 25.909718 | 26.365362 |  |
|  | C9YJE9 |  | NaN | NaN | 23.211621 | 24.408014 | NaN | NaN | 23.086672 | 22.656187 |  |
|  | C9YJF0 |  | 22.353102 | 23.173027 | 23.514004 | 26.399517 | 22.849325 | 24.474693 | 24.757456 | 24.442404 |  |
|  | C9YJG2 |  | 23.173058 | 25.085455 | 23.119812 | 23.879421 | 24.413126 | 24.510387 | 23.606707 | 23.442024 |  |
|  | C9YJH1 |  | 30.381851 | 29.719194 | 30.356155 | 30.684702 | 30.564745 | 29.252302 | 29.892830 | 30.046873 |  |
|  | C9YJH5 |  | 24.214527 | 25.149731 | 23.973513 | 23.509903 | 23.498366 | NaN | 23.608061 | 24.080828 |  |
|  | C9YJI8 |  | 24.825951 | 24.392041 | 24.945166 | 24.320309 | 23.556839 | 23.645594 | 23.543780 | 25.217119 |  |
|  | C9YJJ1 |  | 30.260019 | 29.123072 | 30.153639 | 30.038525 | 30.466455 | 30.823200 | 29.603481 | 29.607807 |  |
|  | C9YJJ3 |  | 27.305738 | 27.190275 | 27.472122 | 27.336796 | 27.387224 | 26.342842 | 26.823473 | 27.224073 |  |
|  | C9YJK5 |  | 32.537834 | 32.227566 | 32.530762 | 32.232323 | 32.502682 | 33.227482 | 32.408390 | 32.158100 |  |
|  | C9YJK7 |  | 31.330900 | 30.651043 | 31.315704 | 31.443520 | 31.584190 | 32.750591 | 31.659533 | 31.204199 |  |
|  | C9YJK8 |  | 24.360313 | 24.045019 | 24.358303 | 23.322649 | 22.778799 | 22.968722 | 23.724840 | 25.605888 |  |
|  | C9YJK9 |  | 26.455523 | 25.924473 | 25.541500 | 25.772837 | 26.219835 | 26.003674 | 23.950470 | 23.928139 |  |
|  | C9YJL7 |  | 29.544952 | 29.381769 | 29.485178 | 29.608036 | 29.853960 | 29.629196 | 29.835615 | 29.812239 |  |
|  | C9YJM0 |  | 22.568691 | NaN | 22.555037 | 22.456336 | NaN | NaN | 23.575424 | 23.456259 |  |
|  | C9YJM4 |  | 28.662853 | 29.056000 | 29.073523 | 29.428043 | 29.106594 | 29.373745 | 25.914972 | 29.138876 |  |
|  | C9YJN0 |  | 26.275244 | 25.881758 | 26.124039 | 27.752960 | 27.547014 | 27.689259 | 26.411316 | 26.503109 |  |
|  | C9YJN5 |  | 31.751108 | 30.183525 | 32.414730 | 32.198208 | 32.206848 | 32.418354 | 32.531830 | 32.175755 |  |
|  | C9YJP4 |  | 27.251240 | 24.293407 | 27.535788 | 27.847269 | 27.188013 | 26.400038 | 27.131338 | 27.224995 |  |
|  | C9YJP8 |  | 27.920254 | 27.516907 | 27.771969 | 27.081518 | 28.046612 | 29.407261 | 27.954214 | 27.629509 |  |
|  | C9YJQ2 |  | 35.328205 | 35.498394 | 35.547283 | 27.725010 | 34.851315 | 25.960867 | 35.863369 | 35.623665 |  |
|  | C9YJQ4;C9YJQ7 |  | 23.807603 | 23.497915 | 23.638977 | NaN | NaN | NaN | 23.772636 | 23.651628 |  |
|  | C9YJQ5 |  | 25.549131 | 24.308207 | 25.172226 | NaN | 24.026817 | NaN | 26.425552 | 25.814701 |  |
|  | C9YJQ8 |  | 25.143709 | 25.271740 | 24.788256 | 23.556604 | 25.157421 | NaN | 24.891247 | 24.827017 |  |
|  | C9YJR1 |  | 24.860933 | 24.732262 | 24.437586 | 23.906923 | 22.966417 | 22.778037 | 22.380504 | 22.689573 |  |
|  | C9YJR4 |  | 22.908409 | 22.887123 | 22.831358 | 23.981112 | 23.211026 | NaN | 22.973447 | 22.907034 |  |
|  | C9YJR6 |  | 25.180218 | 25.372421 | 26.341095 | 26.366230 | 26.030611 | 25.141413 | 25.636385 | 25.205196 |  |
|  | C9YJR7 |  | NaN | NaN | NaN | NaN | NaN | NaN | NaN | 22.441442 |  |
|  | C9YJS3 |  | 25.506094 | 25.144682 | 25.480036 | 24.714605 | 24.643321 | 25.577011 | 25.350636 | 25.636551 |  |
|  | C9YJT1 |  | 25.048599 | 24.122499 | 24.411575 | NaN | 24.741137 | 24.676635 | 23.903526 | NaN |  |
|  | C9YJT9 |  | 24.177521 | 23.380470 | 23.613030 | 23.532553 | 24.478710 | NaN | 23.885765 | 23.810099 |  |
|  | C9YJU0 |  | 25.656683 | 25.969631 | 25.640501 | 25.194302 | 25.662153 | 26.826023 | 25.941385 | 25.478987 |  |
|  | C9YJU6 |  | 25.984919 | 25.451015 | 25.872417 | 25.771528 | 25.974497 | 25.430075 | 25.557423 | 25.762737 |  |
|  | C9YJV2 |  | 26.180977 | 26.949490 | 26.137508 | 27.393948 | 26.311466 | 27.929144 | 27.149672 | 27.220758 |  |
|  | C9YJV8 |  | 24.973993 | 25.086103 | 27.307737 | 27.274088 | 25.038000 | 28.733891 | 22.241009 | 22.417639 |  |
|  | C9YJW6 |  | 25.632120 | 25.763016 | 25.587692 | 25.599348 | 24.965343 | 25.966356 | 25.720959 | 25.651518 |  |
|  | C9YJY3 |  | 24.153137 | 24.832229 | 23.657965 | NaN | 24.003417 | 25.163576 | 24.388638 | 24.139808 |  |
|  | C9YJZ0 |  | 28.606009 | 29.377453 | 28.525633 | 28.704016 | 28.831591 | 28.744837 | 29.011801 | 29.066559 |  |
|  | C9YJZ1 |  | NaN | 23.912691 | NaN | NaN | NaN | NaN | 23.520560 | 23.238020 |  |
|  | C9YK01 |  | 29.739439 | 30.064325 | 29.732712 | 30.717592 | 29.522604 | 27.848225 | 30.921175 | 30.448942 |  |
|  | C9YK02 |  | 28.799032 | 28.521856 | 27.682308 | 28.637409 | 27.342571 | 28.911423 | 28.486349 | 28.051495 |  |
|  | C9YK03 |  | NaN | 25.163116 | 25.987112 | 25.375540 | 25.519894 | 26.684986 | 24.965872 | 25.500544 |  |
|  | C9YK09 |  | 32.359352 | 30.334154 | 32.068562 | 32.009117 | 31.941607 | 30.230089 | 31.589645 | 31.481497 |  |
|  | C9YK10 |  | 31.196714 | 28.922808 | 30.611225 | 30.641342 | 30.989019 | 29.993479 | 30.379995 | 30.526918 |  |
|  | C9YK11 |  | 32.425697 | 30.111727 | 31.886965 | 32.009117 | 32.315327 | 31.114460 | 31.467718 | 31.688583 |  |
|  | C9YK12 |  | 32.144703 | 31.090431 | 32.113434 | 32.350712 | 32.775135 | 32.932220 | 32.243855 | 31.879534 |  |
|  | C9YK13 |  | 34.839821 | 33.839584 | 33.778233 | 34.592400 | 34.561722 | 34.199963 | 34.489330 | 33.922737 |  |
|  | C9YK14 |  | 35.497921 | 33.462463 | 35.372730 | 35.180943 | 35.679996 | 35.857750 | 35.285576 | 34.647854 |  |
|  | C9YK30 |  | 29.043356 | 29.103922 | 29.168850 | 29.468826 | 29.323284 | 30.072655 | 29.413408 | 29.376604 |  |
|  | C9YK38 |  | NaN | 25.047352 | NaN | NaN | NaN | NaN | NaN | NaN |  |
|  | C9YK56 |  | 29.466513 | 31.769117 | 28.999863 | 29.006058 | 28.903400 | 28.465094 | 28.773417 | 29.191429 |  |
|  | C9YK66 |  | 27.307737 | 27.327145 | 26.940781 | 26.924110 | 26.902422 | 27.318293 | 27.689526 | 27.577084 |  |
|  | C9YK67 |  | NaN | 23.799070 | 23.763635 | 23.394405 | 24.177673 | 23.545775 | NaN | NaN |  |
|  | C9YK70 |  | 28.705103 | 29.257683 | 28.805321 | 29.340513 | 28.868471 | 27.780630 | 29.343292 | 28.856173 |  |
|  | C9YK72 |  | 25.008554 | 24.425407 | 24.310566 | 24.434664 | 25.355820 | 24.879982 | 24.971058 | 24.715130 |  |
|  | C9YK74 |  | 27.170546 | 26.173218 | 26.531794 | 26.260942 | 27.353886 | 28.217896 | 26.767746 | 26.705696 |  |
|  | C9YK82 |  | 25.234289 | 25.405647 | 25.327419 | 24.500755 | 25.396204 | 25.795012 | 25.301350 | 25.189560 |  |
|  | C9YK84 |  | 26.943466 | 27.150351 | 26.748808 | 25.892035 | 26.449945 | 26.824688 | 27.105995 | 26.984680 |  |
|  | C9YK89 |  | 23.823764 | 24.894766 | 24.703243 | 25.501545 | 24.386139 | 24.451969 | 24.270493 | 24.237320 |  |
|  | C9YK92 |  | NaN | NaN | NaN | 27.058608 | NaN | NaN | 27.189333 | 26.787683 |  |
|  | C9YK93 |  | 26.150194 | 29.373600 | 27.007742 | 26.609922 | 27.738602 | 26.947708 | 27.005495 | 28.083498 |  |
|  | C9YK97 |  | 25.217009 | 25.126495 | 25.831614 | 25.669012 | NaN | 25.151279 | 25.385506 | 25.225307 |  |
|  | C9YK99 |  | 22.281727 | 22.654919 | 24.013676 | 22.815659 | 21.565092 | 22.287769 | NaN | 21.971092 |  |
|  | C9YKA1 |  | 26.555626 | 26.534157 | 24.947084 | 26.261751 | 26.247171 | 24.273448 | 25.199142 | 25.913170 |  |
|  | C9YKA5 |  | NaN | NaN | NaN | NaN | 22.450294 | NaN | NaN | 22.339493 |  |
|  | C9YKB1 |  | 28.496023 | 28.212618 | 27.392803 | 28.590281 | 27.891222 | 28.215721 | 28.719177 | 28.459202 |  |
|  | C9YKB3 |  | NaN | 25.087399 | 24.236644 | NaN | 24.521931 | 24.818850 | NaN | NaN |  |
|  | C9YKB7 |  | 22.293261 | 22.391445 | 22.283193 | 22.174477 | 22.253265 | 21.778885 | 22.298380 | 22.467695 |  |
|  | C9YKC0 |  | 25.432371 | 26.241653 | NaN | 25.542004 | 25.820088 | 25.914299 | 25.263965 | 25.808291 |  |
|  | C9YKD6 |  | NaN | NaN | 23.333881 | 25.922365 | 22.809195 | 22.816501 | 24.405226 | NaN |  |
|  | C9YKF1 |  | 26.468695 | 25.995352 | 27.459749 | 29.275909 | 27.312420 | 26.329714 | 27.556473 | 27.529696 |  |
|  | C9YKF3 |  | 26.831348 | 28.281889 | 26.995718 | 28.247940 | 26.941118 | 27.407545 | 27.488995 | 27.258268 |  |
|  | C9YKF7 |  | 21.410023 | NaN | 21.871428 | 24.988413 | NaN | NaN | NaN | 22.241593 |  |
|  | C9YKG0 |  | 22.166883 | 21.981257 | 22.490630 | NaN | NaN | NaN | 22.995865 | 23.012754 |  |
|  | C9YKG1 |  | 28.296097 | 28.017546 | 25.353230 | 28.385662 | 28.475992 | 29.083422 | 28.329800 | 28.580322 |  |
|  | C9YKG8 |  | 25.493597 | 25.744761 | 25.455820 | 25.583252 | NaN | NaN | 23.652534 | 24.464634 |  |
|  | C9YKG9 |  | 24.163000 | NaN | 22.463835 | NaN | NaN | NaN | 22.865160 | 23.186148 |  |
|  | C9YKI3 |  | 29.893192 | 30.566923 | 30.176289 | 29.592171 | 29.965542 | 30.250338 | 29.589209 | 30.005577 |  |
|  | C9YKJ1 |  | 27.051548 | 27.617298 | 27.114758 | 26.944473 | 26.805504 | 27.670404 | 27.485083 | 27.339602 |  |
|  | C9YKJ5 |  | 23.794912 | 23.607384 | 25.639921 | 24.344486 | 23.769512 | 23.338106 | 24.450985 | 24.110840 |  |
|  | C9YKK1 |  | 27.557861 | 27.449238 | 28.015526 | 27.424063 | 28.361771 | 28.489759 | 27.826326 | 27.892672 |  |
|  | C9YKK2 |  | 28.143652 | 26.416222 | 29.421276 | 28.051081 | 28.448332 | 29.181820 | 27.689058 | 27.870443 |  |
|  | C9YKL7 |  | 27.099689 | 27.509073 | 24.984507 | 25.746811 | 26.211727 | 26.190935 | 25.742449 | 25.414385 |  |
|  | C9YKL8 |  | 27.044790 | 29.149405 | 26.393032 | 26.454189 | 26.763573 | 27.543036 | 26.281464 | 26.232845 |  |
|  | C9YKM7 |  | 24.124315 | 23.786562 | 24.610542 | 25.499632 | 24.064064 | 23.158772 | 24.178280 | 23.669472 |  |
|  | C9YKN8 |  | 27.857449 | 27.966478 | 28.052324 | 27.964993 | 28.225315 | 26.549925 | 28.095068 | 28.118425 |  |
|  | C9YKP3 |  | 25.446827 | 26.408485 | 24.461889 | 25.322649 | 25.445028 | 25.103095 | 25.491581 | 24.918161 |  |
|  | C9YKQ0 |  | 25.952950 | 27.009880 | 25.662453 | 25.597246 | 25.954370 | NaN | 25.250282 | 25.423998 |  |
|  | C9YKQ1 |  | 23.572536 | 24.731277 | 23.002768 | 23.492405 | 23.286867 | 23.649107 | 24.544746 | 24.135632 |  |
|  | C9YKQ5 |  | 28.582584 | 28.254488 | 27.844997 | 28.242182 | 28.572428 | 28.753216 | 28.254038 | 28.495871 |  |
|  | C9YKQ7 |  | 26.281412 | 25.761570 | 27.333046 | 25.055027 | 25.980001 | 25.406425 | 25.937912 | 26.849836 |  |
|  | C9YKQ9 |  | 24.917252 | 25.809263 | 24.938181 | 24.929853 | 25.007315 | 24.794218 | 25.798080 | 25.411188 |  |
|  | C9YKS0 |  | 25.905777 | 26.416674 | 25.026396 | 24.563372 | 25.987198 | 25.727154 | 25.891685 | 25.746452 |  |
|  | C9YKS3 |  | 27.699024 | 28.692987 | 27.928183 | 27.728256 | 27.466455 | 26.621428 | 26.881104 | 27.598326 |  |
|  | C9YKS4 |  | 26.092569 | 26.729361 | 26.155975 | 26.444901 | 25.773441 | 26.057137 | 26.253155 | 26.195278 |  |
|  | C9YKS8 |  | 32.229031 | 31.175159 | 32.063938 | 31.758991 | 32.028118 | 31.884346 | 31.763035 | 31.483854 |  |
|  | C9YKS9 |  | NaN | NaN | 21.193701 | 21.268595 | NaN | NaN | NaN | NaN |  |
|  | C9YKT3 |  | 27.158848 | 27.860176 | 27.657583 | 27.567904 | 26.919798 | 27.421658 | 27.172932 | 27.478077 |  |
|  | C9YKT6 |  | NaN | NaN | 21.525066 | 21.398640 | NaN | NaN | NaN | NaN |  |
|  | C9YKT8 |  | 24.315598 | 25.494513 | 24.106295 | 24.066774 | 23.716282 | 25.295546 | 23.884460 | 24.429884 |  |
|  | C9YKU3 |  | 24.394472 | 24.975569 | 23.973851 | 24.886929 | 24.451048 | 24.701181 | 24.003332 | 23.962082 |  |
|  | C9YKU5 |  | 30.264053 | 30.755493 | 29.364925 | 29.970350 | 30.680265 | 30.649588 | 30.175337 | 30.350685 |  |
|  | C9YKU7 |  | NaN | NaN | NaN | NaN | 22.876127 | NaN | 22.080584 | 22.133736 |  |
|  | C9YKV4 |  | NaN | NaN | 25.028927 | 23.467445 | NaN | 25.485420 | NaN | 23.153183 |  |
|  | C9YKV5 |  | NaN | NaN | NaN | NaN | NaN | 23.831421 | NaN | NaN |  |
|  | C9YKV6 |  | NaN | 24.424704 | 25.248184 | 24.517492 | NaN | 24.600426 | NaN | 24.305426 |  |
|  | C9YKW5 |  | NaN | NaN | 23.578653 | NaN | NaN | 24.418861 | NaN | NaN |  |
|  | C9YKW6 |  | NaN | NaN | 24.365330 | NaN | NaN | 26.584772 | NaN | 20.918242 |  |
|  | C9YKW9 |  | 23.387850 | 23.982679 | 23.505548 | 23.396498 | 23.742834 | 23.503246 | 23.096821 | 23.114758 |  |
|  | C9YKY6 |  | 26.405388 | 26.095108 | 26.788555 | 26.366730 | 26.401803 | 27.516832 | 26.603428 | 26.200956 |  |
|  | C9YKZ0 |  | 23.869572 | 23.103571 | 23.937597 | 22.687225 | 23.406134 | 23.621801 | 23.378487 | 24.088451 |  |
|  | C9YKZ4 |  | 23.607280 | 23.905455 | 23.536181 | 23.627228 | 23.467747 | 24.244840 | 23.791538 | 23.401714 |  |
|  | C9YKZ8 |  | 24.958364 | 25.199516 | 24.864952 | 24.138880 | 24.713505 | 24.049015 | 23.942884 | 23.567091 |  |
|  | C9YL05 |  | 24.679386 | 24.630621 | 24.131319 | 25.599007 | 23.549663 | 23.955889 | 24.032080 | 24.250826 |  |
|  | C9YL07 |  | NaN | NaN | NaN | NaN | NaN | NaN | 26.653898 | 26.442024 |  |
|  | C9YL30 |  | NaN | NaN | NaN | 22.308165 | NaN | NaN | NaN | NaN |  |
|  | C9YL32 |  | 22.904537 | 23.271133 | 23.443417 | NaN | 22.878094 | NaN | NaN | 22.726753 |  |
|  | C9YL35 |  | 24.101446 | 25.822016 | 24.650150 | 24.995546 | 24.051838 | 25.436062 | 25.005518 | 24.978626 |  |
|  | C9YL46 |  | 27.390099 | 27.344774 | 27.630342 | 26.899660 | 27.478849 | 26.546808 | 27.649313 | 27.755764 |  |
|  | C9YL48 |  | 26.128057 | 26.631176 | 25.995998 | 26.361351 | 25.654335 | 26.498262 | 26.257351 | 26.140417 |  |
|  | C9YL54 |  | 26.421833 | 27.002817 | 27.045103 | 24.888601 | 26.795259 | 26.299517 | 26.778439 | 26.885416 |  |
|  | C9YL55 |  | 24.982113 | NaN | 25.008940 | 25.824858 | 25.668579 | 26.150194 | 24.657038 | 24.624498 |  |
|  | C9YL56 |  | 29.137753 | 23.550290 | 22.047501 | 21.855782 | 27.259794 | NaN | 27.661665 | 27.776934 |  |
|  | C9YL66 |  | 30.634348 | 30.757561 | 30.449730 | 30.635040 | 30.897497 | 29.976372 | 31.445099 | 30.774391 |  |
|  | C9YL71 |  | 32.876591 | 35.757622 | 32.661804 | 32.596039 | 33.200344 | 32.764023 | 32.897030 | 32.624641 |  |
|  | C9YL81 |  | 28.925667 | 27.044373 | 29.326759 | 30.076099 | 29.563419 | 29.893148 | 29.571651 | 29.008198 |  |
|  | C9YL89 |  | 26.419682 | 26.592634 | 26.318552 | 27.265530 | 26.726374 | 27.144722 | 26.900927 | 26.546412 |  |
|  | C9YL92 |  | 22.515810 | 22.041123 | 23.118498 | 22.439312 | NaN | NaN | NaN | NaN |  |
|  | C9YL93 |  | 24.004189 | 23.650314 | 24.007460 | 23.814407 | 23.419699 | NaN | 23.531958 | 23.279304 |  |
|  | C9YL94 |  | 25.844721 | 27.118624 | 25.309872 | 25.976072 | 25.290211 | 26.562250 | 25.613523 | 25.182644 |  |
|  | C9YLA3 |  | NaN | NaN | NaN | NaN | NaN | NaN | 24.353533 | 24.044436 |  |
|  | C9YLA7 |  | 29.495165 | 27.932638 | 27.510206 | 22.070448 | 28.869177 | 25.902559 | 29.677965 | 29.572283 |  |
|  | C9YLA8 |  | 24.046352 | NaN | 25.194416 | 28.451677 | 23.929583 | 23.887247 | 25.988348 | 24.010435 |  |
|  | C9YLB0 |  | 29.215931 | 28.848701 | 27.635057 | 26.463760 | 27.703981 | 27.981689 | 24.943958 | 26.664923 |  |
|  | C9YLB9 |  | 23.795507 | 25.837112 | 24.546631 | 24.300756 | 23.720568 | 23.261359 | 24.282915 | 24.416416 |  |
|  | C9YLC1 |  | 34.658318 | 36.934517 | 34.532875 | 34.627720 | 34.972542 | 35.227661 | 34.918831 | 34.487354 |  |
|  | C9YLC3 |  | 26.457825 | 26.956154 | 25.912714 | 26.442673 | 26.688457 | 24.130848 | 27.290878 | 26.981634 |  |
|  | C9YLC6 |  | 24.595312 | 25.435999 | 25.023691 | 25.874786 | 24.895042 | 25.617424 | 25.769537 | 25.207880 |  |
|  | C9YLD3 |  | 25.598183 | 23.191128 | 25.251764 | 24.673035 | 25.160080 | 24.863298 | 24.908939 | 25.243181 |  |
|  | C9YLD4 |  | 24.083914 | NaN | 23.392572 | 23.613808 | 23.852339 | NaN | 23.909580 | 23.904262 |  |
|  | C9YLE4 |  | NaN | 27.765345 | 27.169399 | 28.011053 | 28.401415 | 27.152769 | 27.577660 | 27.950714 |  |
|  | C9YLE5 |  | NaN | 26.660034 | 26.527430 | 27.214657 | 27.675999 | 26.314444 | 26.759895 | 27.026121 |  |
|  | C9YLE7 |  | 27.273199 | 26.545837 | 26.120800 | 26.882620 | 27.216045 | 26.320034 | 27.193758 | 26.868206 |  |
|  | C9YLE9 |  | 26.287729 | 26.671146 | 25.002602 | 26.406458 | 25.326220 | NaN | 25.201536 | 24.832870 |  |
|  | C9YLF1 |  | 25.724997 | 25.221550 | 23.714920 | 25.679815 | 25.462919 | 25.660332 | 25.515690 | 26.048807 |  |
|  | C9YLF3 |  | 25.115412 | 26.713978 | 25.085739 | 24.938225 | 25.083630 | 25.082453 | 25.211857 | 24.833498 |  |
|  | C9YLF4 |  | 25.026312 | 25.157654 | 24.862684 | 24.446133 | 23.829259 | 22.831846 | 24.711458 | 24.467808 |  |
|  | C9YLG4 |  | NaN | NaN | NaN | NaN | NaN | NaN | 25.967148 | 26.317535 |  |
|  | C9YLI9 |  | 22.172462 | NaN | NaN | 23.632896 | NaN | NaN | NaN | NaN |  |
|  | C9YLL2 |  | NaN | NaN | NaN | NaN | NaN | NaN | 26.306538 | 25.841005 |  |
|  | C9YLL4 |  | 23.319620 | 26.805996 | 22.636772 | 23.630787 | 21.553608 | 22.733636 | 23.855764 | 23.850241 |  |
|  | C9YLM1 |  | NaN | NaN | 22.946674 | 22.674179 | 22.381967 | NaN | NaN | NaN |  |
|  | C9YLM6 |  | 25.787607 | 26.051775 | NaN | 25.741549 | 25.323370 | NaN | 25.606594 | 25.142191 |  |
|  | C9YLN4 |  | 29.002682 | 30.658808 | 27.651077 | 28.381872 | NaN | 27.192160 | 28.941454 | 28.967550 |  |
|  | C9YLP5 |  | 23.144711 | 23.010246 | 22.889929 | 23.215630 | NaN | NaN | 24.138447 | 23.898460 |  |
|  | C9YLQ7 |  | 29.332212 | 25.935575 | 28.270662 | 28.606607 | 28.920338 | 28.241999 | 28.107094 | 27.791290 |  |
|  | C9YLR3 |  | 21.495459 | NaN | 22.163246 | 21.952658 | NaN | NaN | NaN | NaN |  |
|  | C9YLR7 |  | 26.107632 | 28.064659 | 26.044706 | 27.148314 | 25.827719 | 26.005154 | 25.803782 | 25.200190 |  |
|  | C9YLR8 |  | 30.010920 | 31.967537 | 30.226763 | 30.004772 | 30.018633 | 28.807533 | 29.607279 | 30.151463 |  |
|  | C9YLS9 |  | 25.797859 | 25.897676 | 24.995071 | 27.608301 | 25.308554 | 25.217155 | 27.058788 | 26.573114 |  |
|  | C9YLT0 |  | 29.770662 | 29.976919 | 29.639153 | 30.635990 | 29.746937 | 29.962097 | 30.536955 | 30.408112 |  |
|  | C9YLT1 |  | 27.972187 | 27.832315 | 26.544260 | 27.856085 | 27.151125 | 27.266512 | 27.785751 | 27.463808 |  |
|  | C9YLT2 |  | NaN | 22.566965 | 22.688187 | 22.081705 | 22.551592 | 22.089712 | 22.363245 | 22.128773 |  |
|  | C9YLT4 |  | 22.791881 | 22.962294 | 23.159203 | 22.518376 | 22.650763 | NaN | 23.230608 | NaN |  |
|  | C9YLT5 |  | 24.661665 | 25.207283 | 24.886463 | 25.097481 | 24.700071 | 24.459578 | 23.403797 | 23.804422 |  |
|  | C9YLU7 |  | 26.051548 | 25.166338 | 26.151493 | 25.519833 | 25.795160 | 26.179478 | 25.977491 | 25.696705 |  |
|  | C9YLU8 |  | NaN | NaN | NaN | NaN | NaN | NaN | 22.583412 | 24.303894 |  |
|  | C9YLV5 |  | NaN | NaN | NaN | NaN | NaN | 26.570395 | NaN | NaN |  |
|  | C9YLV7 |  | 26.086590 | 26.528549 | 24.806759 | 25.467342 | 25.309525 | 23.664814 | 24.933821 | 25.398718 |  |
|  | C9YLV8 |  | 24.707514 | 25.852053 | 24.698217 | 23.968424 | 24.850479 | 25.388670 | 24.438120 | 25.014484 |  |
|  | C9YLW8 |  | 25.516861 | 26.672226 | 25.520733 | 25.570251 | 25.898829 | 25.450922 | 25.281322 | 25.144255 |  |
|  | C9YLX0 |  | 24.278172 | 24.498871 | NaN | 24.217415 | NaN | 24.737880 | 25.180824 | 25.623884 |  |
|  | C9YLX7 |  | 24.481546 | 24.666656 | 24.395714 | 25.529516 | 24.178053 | NaN | 24.946012 | 24.944138 |  |
|  | C9YLY2 |  | 24.656328 | 25.427999 | 24.303963 | 24.614422 | 24.506094 | 25.228724 | 24.744940 | 24.502882 |  |
|  | C9YLY5 |  | 30.061110 | 32.763233 | 29.406309 | 30.201513 | 30.388334 | 30.055561 | 30.066637 | 29.595909 |  |
|  | C9YLY6 |  | 22.615490 | 27.597403 | 23.594201 | NaN | 23.933731 | NaN | 24.147326 | 23.820749 |  |
|  | C9YLY8 |  | 31.571226 | 31.841837 | 31.601429 | 31.200460 | 31.468155 | 32.572220 | 30.728256 | 31.190371 |  |
|  | C9YLY9 |  | 25.357229 | 25.407173 | 25.575829 | 26.747015 | 25.415255 | 26.365646 | 25.298765 | 25.657473 |  |
|  | C9YLZ2 |  | NaN | 26.359409 | NaN | 24.256739 | 24.486034 | NaN | NaN | NaN |  |
|  | C9YLZ7 |  | 24.133282 | 23.817142 | 24.363592 | 24.257172 | 24.939661 | 23.208185 | 26.734280 | 26.409569 |  |
|  | C9YM07 |  | 25.157885 | 24.275264 | 25.269136 | 24.756235 | NaN | 25.487600 | 24.368330 | 24.243362 |  |
|  | C9YM08 |  | 27.084158 | 26.876427 | 26.385332 | 26.163288 | 26.747145 | 26.177748 | 26.610344 | 26.398701 |  |
|  | C9YM13 |  | 23.290949 | 25.510960 | 25.092609 | 24.422523 | 24.415514 | NaN | 24.563139 | 24.215021 |  |
|  | C9YM14 |  | 24.385679 | 23.867800 | 25.714422 | 25.447899 | 25.181242 | 25.127625 | 24.707041 | 24.710197 |  |
|  | C9YM24 |  | 23.322360 | 24.454252 | 22.868725 | 23.332243 | 22.771378 | 23.539076 | 23.943869 | 23.598553 |  |
|  | C9YM30 |  | NaN | NaN | 21.747978 | 22.581736 | 21.858328 | NaN | NaN | 21.859314 |  |
|  | C9YM40 |  | 28.815098 | 30.552429 | 29.114237 | 29.540636 | 28.745991 | 28.307781 | 28.888264 | 29.585756 |  |
|  | C9YM42 |  | 27.822319 | 26.929989 | 26.731434 | 26.663296 | 26.996365 | 27.909306 | 27.886230 | 27.843140 |  |
|  | C9YM59 |  | 23.487997 | 24.913193 | 24.324436 | 23.890133 | 23.761520 | 23.306670 | 22.910515 | 22.904836 |  |
|  | C9YM65 |  | 24.262054 | 25.169783 | 23.880850 | 25.143166 | 24.838747 | 25.269566 | 25.053453 | NaN |  |
|  | C9YM70 |  | NaN | 23.304102 | NaN | NaN | 23.332516 | 24.950825 | NaN | NaN |  |
|  | C9YM72 |  | 25.584801 | 25.929853 | 25.196556 | 25.303963 | 25.236700 | NaN | 25.224350 | 25.108809 |  |
|  | C9YM76 |  | 28.685989 | 28.452894 | 27.411995 | 28.009506 | 27.504154 | 26.961132 | 26.941229 | 27.104197 |  |
|  | C9YM84 |  | 23.813038 | 22.607836 | 23.940666 | 24.769563 | 23.737463 | NaN | 24.080259 | 23.971401 |  |
|  | C9YM87 |  | 29.488764 | 27.837135 | 29.793478 | 29.145208 | 29.742767 | 28.909763 | 29.281269 | 29.471077 |  |
|  | C9YM88 |  | 22.609596 | 23.127735 | 24.177748 | 23.944227 | 25.017078 | 22.909567 | 23.540970 | 24.673899 |  |
|  | C9YM90 |  | 25.788280 | 25.377195 | 24.598780 | 27.347816 | 25.306713 | 23.816415 | 26.801069 | 26.413416 |  |
|  | C9YM93 |  | NaN | 23.177567 | 23.630121 | 22.970303 | 24.107332 | NaN | 24.499905 | 24.305147 |  |
|  | C9YM97 |  | 21.904776 | 22.105112 | NaN | NaN | NaN | NaN | 22.870701 | 22.199314 |  |
|  | C9YM99 |  | NaN | NaN | NaN | NaN | NaN | NaN | 22.646408 | 22.651052 |  |
|  | C9YMA5 |  | 22.971514 | 23.637104 | 23.264706 | 22.988342 | NaN | NaN | 22.912783 | 23.039541 |  |
|  | C9YMB2 |  | 26.848881 | 31.131977 | 26.997227 | 27.303650 | 26.857449 | 26.027262 | 27.519459 | 27.407141 |  |
|  | C9YMC4 |  | 25.907198 | 25.831131 | 25.594770 | 26.258053 | 24.686831 | NaN | 25.797932 | 25.376003 |  |
|  | C9YMC5 |  | 28.005495 | 25.184536 | 27.823778 | 27.082737 | 25.097441 | 25.079039 | 24.466440 | 25.223541 |  |
|  | C9YMJ4 |  | NaN | NaN | 23.798576 | NaN | 23.838266 | NaN | 24.392836 | 24.283833 |  |
|  | C9YMK2 |  | 24.141006 | 24.478647 | 25.281748 | 26.525087 | 24.145771 | 25.167006 | 24.246483 | NaN |  |
|  | C9YMK3 |  | NaN | NaN | NaN | 23.960138 | NaN | NaN | NaN | NaN |  |
|  | C9YML0 |  | 26.675594 | 28.531927 | 27.476688 | 26.602579 | 25.452713 | NaN | 26.241091 | 26.820677 |  |
|  | C9YMP8 |  | 27.863901 | 27.624916 | 27.983213 | 28.731369 | 27.864138 | 27.607737 | 28.150688 | 27.782318 |  |
|  | C9YMP9 |  | 25.515146 | NaN | 27.307999 | 29.952549 | 26.820433 | 27.558884 | 27.519009 | 26.386725 |  |
|  | C9YMQ5 |  | 23.154636 | NaN | 25.032925 | 28.386362 | 24.662643 | 25.192219 | 25.635279 | 24.188881 |  |
|  | C9YMQ7 |  | 27.837677 | 29.740597 | 27.783941 | 27.745222 | 27.743488 | 27.596905 | 27.151415 | 27.164133 |  |
|  | C9YMT4 |  | 29.470844 | 27.998035 | 29.263851 | 28.834154 | 29.527311 | 28.606890 | 29.066971 | 28.697832 |  |
|  | C9YMT6 |  | 29.569788 | 30.076736 | 29.396482 | 29.881599 | 29.603374 | 30.308887 | 29.939016 | 29.955462 |  |
|  | C9YMT7 |  | 27.879936 | 28.181961 | 27.782818 | 27.620869 | 27.390427 | 27.835691 | 27.302517 | 27.737570 |  |
|  | C9YMT8 |  | 22.299778 | 22.689945 | 23.741188 | 23.501425 | 22.560995 | 24.042265 | 22.299807 | 22.669472 |  |
|  | C9YMU3 |  | 27.223614 | 27.437302 | 26.614843 | 26.672226 | 27.015846 | 27.135160 | 27.409893 | 27.192253 |  |
|  | C9YMU5 |  | 28.222048 | 28.193146 | 27.889715 | 27.675123 | 28.091288 | 28.156342 | 28.496784 | 28.320318 |  |
|  | C9YMV9 |  | 29.297520 | 28.266333 | 29.510073 | 30.323242 | 30.756607 | 22.802071 | 30.010254 | 29.342339 |  |
|  | C9YMW6 |  | 24.365403 | NaN | NaN | NaN | NaN | 25.528921 | NaN | NaN |  |
|  | C9YMX4 |  | 26.462450 | 26.537315 | 27.020197 | 27.177027 | 26.621286 | 27.395420 | 25.916796 | 26.404268 |  |
|  | C9YMY1 |  | 24.337425 | 22.880972 | 24.674168 | 24.019201 | 24.203070 | 24.352154 | 23.559988 | 23.634668 |  |
|  | C9YMY2 |  | NaN | 23.786448 | 23.750931 | 24.699011 | 23.244074 | NaN | 24.695883 | 23.914244 |  |
|  | C9YMY3 |  | 25.175620 | 26.226429 | 25.283232 | 25.497805 | 25.219352 | 26.331406 | 25.113506 | 25.552917 |  |
|  | C9YMZ8 |  | 22.355349 | NaN | 23.192362 | 22.844851 | 22.531885 | NaN | NaN | 21.131445 |  |
|  | C9YN06 |  | 22.653516 | 23.869854 | 23.154095 | 23.644934 | 22.885672 | 24.793722 | 23.148876 | 23.598553 |  |
|  | C9YN08 |  | 25.257927 | 25.103497 | 25.107014 | 26.073042 | 25.183704 | 24.098769 | 24.694395 | 24.985201 |  |
|  | C9YN09 |  | 26.228394 | 29.738714 | 30.287140 | 30.703058 | 30.842960 | 27.672091 | 27.780254 | 21.857771 |  |
|  | C9YN12 |  | 27.737055 | 27.201723 | 28.981798 | 27.347395 | 27.315964 | 26.716092 | 25.097923 | 25.469299 |  |
|  | C9YN24 |  | 24.149109 | 25.018946 | 25.262808 | 24.408274 | 25.261948 | 24.670877 | 24.843500 | 24.980198 |  |
|  | C9YN32 |  | NaN | NaN | 21.297266 | 21.480892 | NaN | NaN | NaN | NaN |  |
|  | C9YN55 |  | 23.897352 | 24.007381 | 23.493994 | 23.913786 | 23.727335 | 24.215046 | NaN | NaN |  |
|  | C9YN58 |  | 28.713453 | 26.940557 | 26.997980 | 28.551788 | 28.912766 | 28.999783 | 27.827719 | 27.338837 |  |
|  | C9YN65 |  | NaN | 26.305096 | NaN | NaN | 21.797212 | 22.049862 | 23.384428 | 22.843317 |  |
|  | C9YN66 |  | NaN | NaN | 23.438387 | 22.956190 | 22.859200 | NaN | 22.239612 | 22.453825 |  |
|  | C9YN69 |  | NaN | NaN | NaN | 25.675245 | 21.512486 | NaN | 21.873051 | NaN |  |
|  | C9YN75 |  | 29.797750 | 29.705368 | 29.792717 | 31.210192 | 29.306000 | 30.714239 | 31.170450 | 30.595127 |  |
|  | C9YN85 |  | 27.869089 | 28.547125 | 28.101143 | 27.181772 | 27.468088 | 27.604843 | 27.306261 | 27.652121 |  |
|  | C9YN86 |  | 25.072878 | 25.786961 | 25.330090 | 25.991140 | 24.967192 | 24.358706 | 25.003845 | 25.367865 |  |
|  | C9YN87 |  | NaN | 23.655563 | 24.318090 | 23.138658 | 23.229904 | NaN | 23.162954 | 23.153394 |  |
|  | C9YN99 |  | 30.048302 | 29.688393 | 29.744659 | 30.326782 | 30.372749 | 30.837030 | 30.289671 | 29.758419 |  |
|  | C9YNA5 |  | 26.185993 | 23.610549 | 24.938675 | 24.683140 | 24.602162 | 24.793325 | 23.578394 | 23.543095 |  |
|  | C9YNA9 |  | 30.122300 | 27.188673 | 32.269775 | 29.546757 | 28.427504 | NaN | 28.080226 | 29.499050 |  |
|  | C9YNB3 |  | 22.346813 | 26.696043 | 22.770319 | NaN | NaN | NaN | NaN | 21.766846 |  |
|  | C9YNB5 |  | 23.378883 | NaN | 22.244073 | 22.403978 | 21.788952 | 22.603239 | 21.753973 | 22.129276 |  |
|  | C9YNC1 |  | NaN | NaN | 27.432291 | 26.961573 | 26.703981 | 29.094362 | 26.879118 | 27.787497 |  |
|  | C9YNC4 |  | NaN | 23.263990 | 28.377659 | 29.147102 | 28.123367 | 30.390284 | 28.289560 | 29.268097 |  |
|  | C9YNE0 |  | 27.292635 | 27.535788 | 27.604630 | 27.273554 | 27.349758 | 26.398277 | 27.315100 | 27.748041 |  |
|  | C9YNE2 |  | 30.869604 | 31.018965 | 31.228313 | 31.346624 | 30.893164 | 31.400295 | 31.544346 | 31.314896 |  |
|  | C9YNG1 |  | 23.515930 | 21.770117 | 24.289190 | 24.953003 | 23.174734 | NaN | 23.367731 | 23.865141 |  |
|  | C9YNG5 |  | 22.121133 | 21.806051 | 22.728893 | 22.161070 | 21.845253 | NaN | NaN | NaN |  |
|  | C9YNI0 |  | 25.780518 | 25.417381 | 25.894650 | 25.631260 | 25.733295 | 28.513447 | 25.077164 | 25.283127 |  |
|  | C9YNI5 |  | 27.578739 | 27.012865 | 27.235258 | 27.666414 | 27.925922 | 27.855549 | 27.678553 | 27.760149 |  |
|  | C9YNJ2 |  | 23.476213 | 24.202171 | 23.145195 | 24.603655 | 22.704177 | 24.675514 | 23.890690 | NaN |  |
|  | C9YNJ9 |  | 26.396938 | 25.791365 | 26.289930 | 25.976246 | 25.921049 | 25.330090 | 25.757328 | 25.585718 |  |
|  | C9YNK1 |  | 30.249207 | 30.508337 | 30.080681 | 31.743345 | 30.691706 | 31.554810 | 31.641384 | 30.973295 |  |
|  | C9YNK3 |  | 26.977055 | 28.845924 | 27.884369 | 27.703785 | 26.987936 | 27.170069 | 27.801378 | 27.923998 |  |
|  | C9YNK8 |  | 23.660469 | 24.130692 | 24.720201 | 23.732624 | 23.151016 | NaN | 23.297121 | 23.740675 |  |
|  | C9YNK9 |  | 29.458048 | 29.971310 | 29.403671 | 29.511299 | 29.459358 | 21.756092 | 29.777576 | 29.842272 |  |
|  | C9YNN9 |  | 25.130339 | 24.947798 | 25.258825 | 22.931479 | 26.109844 | 23.810293 | 24.659107 | 24.461763 |  |
|  | C9YNP2 |  | 23.930576 | 23.271133 | 24.545984 | 23.476908 | 24.439743 | NaN | 23.552742 | 23.754503 |  |
|  | C9YNP6 |  | 23.684103 | 24.567207 | 24.750879 | 23.963669 | 23.332653 | 24.121868 | NaN | 22.843893 |  |
|  | C9YNP8 |  | 25.769133 | 25.260477 | 25.314720 | 24.924156 | 25.551804 | 26.517899 | 25.320068 | 25.718765 |  |
|  | C9YNP9 |  | 28.456110 | 29.048639 | 28.155521 | 27.928015 | 28.310253 | 28.284760 | 27.901848 | 28.164421 |  |
|  | C9YNQ0;C9YRS8 |  | 27.071630 | 26.941677 | 27.005817 | 26.347868 | 26.875841 | 26.483593 | 26.472137 | 26.816044 |  |
|  | C9YNQ7 |  | 30.691624 | 30.539083 | 30.386074 | 30.442530 | 30.707590 | 31.543285 | 30.134426 | 29.830835 |  |
|  | C9YNR3 |  | 30.525614 | 30.896271 | 30.999876 | 29.774830 | 30.128786 | 29.285553 | 30.018898 | 30.560379 |  |
|  | C9YNR6 |  | 27.796806 | 25.941454 | 29.419107 | 29.019854 | 27.873028 | 23.445189 | 29.165068 | 29.501955 |  |
|  | C9YNR7 |  | 25.000584 | NaN | 25.003117 | 24.733709 | 25.872557 | NaN | 25.286831 | 26.072674 |  |
|  | C9YNR9 |  | 31.427723 | 28.882154 | 32.424324 | 32.483158 | 32.206501 | 29.664940 | 32.591610 | 32.421318 |  |
|  | C9YNS0 |  | 29.600153 | 27.011267 | 30.771324 | 30.712273 | 30.246492 | 27.684383 | 30.770222 | 30.606997 |  |
|  | C9YNS6 |  | 27.946873 | 26.489897 | 29.841656 | 30.236261 | 28.860857 | 28.576506 | 28.642616 | 28.273510 |  |
|  | C9YNS7 |  | 29.376831 | 27.879469 | 31.136690 | 31.574974 | 30.112844 | 29.891455 | 29.859701 | 29.247013 |  |
|  | C9YNS8 |  | 22.343337 | NaN | 25.137001 | 24.335722 | 22.783888 | 23.564725 | 22.743122 | 24.196405 |  |
|  | C9YNT0 |  | 26.441660 | 25.464447 | 27.892149 | 28.336199 | 26.045269 | 26.957262 | 27.078875 | 27.080503 |  |
|  | C9YNT1 |  | 25.630898 | NaN | 27.633533 | 28.237175 | 27.164038 | 27.043644 | 26.041555 | 25.353565 |  |
|  | C9YNT2 |  | 23.494789 | NaN | 24.302151 | 24.780416 | 23.085470 | 23.705355 | 23.507729 | 24.604673 |  |
|  | C9YNU0 |  | 27.360180 | 27.623522 | 26.749960 | 26.642099 | 27.566525 | 26.726114 | 26.538233 | 26.632841 |  |
|  | C9YNU1 |  | 23.029669 | 23.415639 | NaN | 23.306608 | 22.856333 | NaN | 23.006571 | 22.660185 |  |
|  | C9YNU4 |  | 28.950798 | 28.370277 | 28.622614 | 29.462950 | 29.548302 | 28.157402 | 28.648628 | 28.447821 |  |
|  | C9YNV5 |  | 24.969917 | 24.033892 | 25.942684 | 24.319138 | 25.455162 | 24.762533 | 26.127958 | 26.686056 |  |
|  | C9YNV6 |  | 26.937979 | 24.234225 | 27.466066 | 23.379886 | 27.444269 | 23.771233 | 28.305826 | 28.484352 |  |
|  | C9YNV7 |  | 26.609499 | 23.925695 | 27.014996 | 23.452745 | 26.007359 | 22.929276 | 26.705170 | 27.570511 |  |
|  | C9YNW2 |  | NaN | NaN | 24.788952 | 25.424288 | 25.357901 | NaN | 25.825659 | 25.284397 |  |
|  | C9YNW5 |  | 30.300293 | 24.570570 | NaN | 22.364983 | 30.153156 | 31.608849 | 29.664551 | 29.487173 |  |
|  | C9YNX3 |  | 29.578865 | 29.486675 | 29.387636 | 29.747320 | 29.631470 | 29.854584 | 30.089420 | 30.049990 |  |
|  | C9YNX9 |  | 22.492798 | 23.818605 | 23.006826 | 22.796001 | 22.602000 | 22.665096 | 22.529486 | 23.067940 |  |
|  | C9YNY0 |  | 28.290396 | 28.469873 | 28.280386 | 28.166386 | 28.474754 | 28.333002 | 28.370235 | 28.367947 |  |
|  | C9YNY3 |  | 23.644329 | 24.278242 | 23.318518 | 23.306481 | 23.666656 | NaN | 23.207886 | 23.810277 |  |
|  | C9YNY5 |  | 28.534378 | 28.479004 | 28.115898 | 27.860235 | 28.289911 | 27.907532 | 27.954491 | 27.993183 |  |
|  | C9YP13 |  | 27.169018 | 27.510658 | 27.216879 | 27.270706 | 26.222509 | 26.832918 | 26.247007 | 26.330141 |  |
|  | C9YP25 |  | 27.787184 | 28.141705 | 27.765976 | 27.177502 | 27.324230 | 27.445059 | 27.066610 | 27.354053 |  |
|  | C9YP27 |  | 30.598503 | 29.981144 | 30.740934 | 29.177336 | 30.294279 | 26.989777 | 29.542833 | 30.094967 |  |
|  | C9YP32 |  | 22.627789 | NaN | 23.411963 | 22.770580 | 23.203861 | 23.271420 | NaN | NaN |  |
|  | C9YP33 |  | 26.291073 | 24.897121 | 26.753662 | 26.593348 | 26.823595 | 27.815800 | 24.501425 | 24.903894 |  |
|  | C9YP35 |  | NaN | NaN | 22.749888 | 22.621443 | NaN | 22.841860 | NaN | 21.545899 |  |
|  | C9YP39 |  | 26.733633 | 27.280209 | 26.515179 | 26.953825 | 26.496099 | 26.165092 | 26.211393 | 26.583481 |  |
|  | C9YP41 |  | 29.943914 | 30.272755 | 30.247963 | 30.414074 | 29.425684 | 30.169973 | 29.348577 | 29.816898 |  |
|  | C9YP49 |  | NaN | NaN | NaN | 23.438602 | NaN | NaN | NaN | NaN |  |
|  | C9YP56 |  | 25.760454 | 29.261274 | 25.246700 | 22.093184 | 24.802378 | 23.788435 | 22.414804 | 23.294458 |  |
|  | C9YP57 |  | 23.618069 | 24.894302 | 24.635445 | 23.939302 | NaN | 22.745904 | 23.303501 | 23.284185 |  |
|  | C9YP60 |  | 24.518633 | 24.142685 | 22.744148 | 24.255587 | 24.807840 | 25.391197 | 23.040344 | 23.072821 |  |
|  | C9YP63 |  | 22.779184 | 23.330403 | 22.740826 | 22.509155 | 23.117272 | 24.099372 | 23.149250 | 23.548044 |  |
|  | C9YP74 |  | 26.831106 | 25.652969 | 25.968445 | 25.686991 | 25.786636 | 24.045353 | 25.442909 | 25.703455 |  |
|  | C9YP75 |  | 24.214602 | 24.559816 | 24.336063 | 24.430840 | 24.596506 | NaN | 24.209106 | 23.987633 |  |
|  | C9YP77 |  | 25.647545 | 27.507940 | 25.984375 | 26.727413 | 26.017822 | 25.703638 | 26.521122 | 26.426161 |  |
|  | C9YP81 |  | 26.665600 | 24.842588 | 25.451078 | 25.643835 | 26.169725 | 26.161041 | 25.469206 | 25.351208 |  |
|  | C9YP88 |  | 24.445564 | 23.258825 | 24.745195 | 24.236517 | 24.254795 | 23.997400 | 23.910221 | 24.276539 |  |
|  | C9YP92 |  | 31.922878 | 31.945169 | 31.708042 | 32.035645 | 32.128323 | 32.399097 | 32.152462 | 31.664711 |  |
|  | C9YP93 |  | 25.865471 | 24.324780 | 24.383373 | 25.980371 | 26.279837 | 25.402626 | 26.362957 | 26.140301 |  |
|  | C9YP98 |  | NaN | NaN | 24.891199 | 23.063051 | NaN | NaN | 22.519821 | 22.734207 |  |
|  | C9YPA5 |  | 30.981213 | 31.063232 | 30.587725 | 30.479813 | 30.386999 | 30.918406 | 32.306229 | 32.271614 |  |
|  | C9YPB3 |  | NaN | 27.068253 | NaN | NaN | NaN | 24.188881 | 23.035938 | 22.887680 |  |
|  | C9YPC0 |  | 29.363462 | 29.187588 | 29.352266 | 29.487347 | 29.538473 | 27.947931 | 29.391922 | 29.522867 |  |
|  | C9YPC3 |  | 27.077755 | 27.173885 | 26.856976 | 27.149769 | 27.276575 | 27.394112 | 27.502335 | 27.543110 |  |
|  | C9YPC9 |  | 23.873989 | 24.019115 | 23.580034 | 23.787558 | 23.475498 | 23.513523 | 23.533127 | 23.600368 |  |
|  | C9YPD3 |  | 24.675837 | 25.958080 | 25.419729 | 25.872887 | 24.478031 | 25.554996 | 25.189333 | 25.344486 |  |
|  | C9YPD4 |  | 24.034117 | 24.286514 | NaN | NaN | 24.416544 | NaN | NaN | 24.135710 |  |
|  | C9YPE4 |  | 29.982098 | 27.878008 | 29.350412 | 29.511677 | 29.894638 | 30.264948 | 29.314863 | 29.320662 |  |
|  | C9YPE6 |  | 25.270706 | 25.193663 | 24.767150 | 25.020813 | 24.332516 | 27.808638 | 24.631121 | 25.272238 |  |
|  | C9YPE7 |  | 29.642305 | 29.281181 | 29.190535 | 28.855341 | 29.257549 | 29.078213 | 28.655230 | 29.185345 |  |
|  | C9YPF0 |  | NaN | 23.374512 | NaN | 22.017730 | NaN | NaN | NaN | 21.830533 |  |
|  | C9YPF7 |  | 27.100992 | 28.042547 | 26.951715 | 26.603994 | 26.909649 | 27.605055 | 27.157402 | 27.094866 |  |
|  | C9YPG1 |  | 25.405128 | 25.829100 | 25.499113 | 25.402918 | 25.192987 | 25.799540 | 25.170776 | 25.370161 |  |
|  | C9YPG2 |  | 27.057550 | 27.110483 | 26.696440 | 26.840382 | 27.094564 | 26.846970 | 26.837858 | 27.110880 |  |
|  | C9YPG4 |  | NaN | NaN | 24.471224 | 24.778432 | NaN | 24.079157 | NaN | 23.462887 |  |
|  | C9YPH3 |  | 25.995653 | 25.210743 | 26.026628 | 25.756922 | 25.791241 | 26.379015 | 25.043936 | 25.088774 |  |
|  | C9YPH7 |  | NaN | NaN | NaN | NaN | NaN | 24.331287 | 26.690458 | 26.436539 |  |
|  | C9YPH8 |  | NaN | NaN | NaN | NaN | NaN | 23.430458 | 28.893858 | 28.589638 |  |
|  | C9YPH9 |  | 27.931005 | 28.323929 | 27.812988 | 25.337322 | 27.735506 | 26.254172 | 23.017363 | NaN |  |
|  | C9YPI2 |  | 32.192459 | 33.299801 | 31.800623 | 33.127480 | 33.045425 | 32.620407 | 33.537048 | 33.102322 |  |
|  | C9YPI9 |  | 23.186707 | 24.120682 | 26.023268 | 24.032125 | 22.975210 | 23.185074 | 23.695192 | 24.125183 |  |
|  | C9YPJ3 |  | 23.964448 | 24.976706 | 24.213415 | NaN | 24.230154 | 24.235069 | 24.170891 | 23.962788 |  |
|  | C9YPJ6 |  | 28.255524 | 26.933258 | 28.580393 | 27.434042 | 28.521444 | 29.089926 | 29.102720 | 28.869648 |  |
|  | C9YPJ7 |  | 28.015526 | 26.941790 | 28.137947 | 27.183193 | 28.268476 | 28.700712 | 29.185604 | 29.034384 |  |
|  | C9YPK2 |  | 24.395321 | 23.835082 | NaN | NaN | 23.742503 | NaN | 23.949223 | 24.802525 |  |
|  | C9YPL9 |  | 27.387470 | 28.548595 | NaN | 27.887451 | 27.751492 | NaN | 27.280386 | 27.741432 |  |
|  | C9YPM0 |  | 26.784441 | 26.784691 | 27.222692 | 27.249975 | 26.571611 | 26.065523 | 26.082657 | 26.540482 |  |
|  | C9YPM3 |  | 24.820944 | 24.113895 | 24.457762 | 25.300371 | 24.292494 | 25.222914 | 23.286402 | 23.050642 |  |
|  | C9YPN0 |  | NaN | NaN | 23.335227 | 24.541796 | NaN | 22.608175 | NaN | NaN |  |
|  | C9YPP5 |  | 32.142849 | 33.317028 | 32.045605 | 32.039570 | 32.762005 | 33.047001 | 32.008751 | 31.839211 |  |
|  | C9YPQ5 |  | 32.721325 | 33.459534 | 32.163479 | 32.492882 | 32.490349 | 30.981417 | 32.478920 | 32.658661 |  |
|  | C9YPQ6 |  | 32.378807 | 32.042770 | 32.374615 | 32.931286 | 32.820065 | 33.100475 | 32.207401 | 32.012020 |  |
|  | C9YPR5 |  | 26.056559 | 25.445690 | 25.115253 | 24.990448 | 25.696997 | 25.673170 | 25.199554 | 25.726711 |  |
|  | C9YPS4 |  | 25.071733 | 25.398947 | 25.748680 | 25.608118 | 25.560604 | 25.842636 | 25.811102 | 26.491718 |  |
|  | C9YPS8 |  | 27.017014 | 26.936182 | 26.921616 | 26.797239 | 27.415224 | NaN | 27.113466 | 27.487309 |  |
|  | C9YPT6 |  | 27.853588 | 27.974213 | 27.217896 | 27.726374 | 27.410460 | 27.589495 | 28.543627 | 27.966038 |  |
|  | C9YPU1 |  | 26.895737 | 28.571596 | 27.371025 | 27.565073 | 26.666008 | 27.902536 | 27.485390 | 27.310686 |  |
|  | C9YPU3 |  | 24.770168 | 25.912302 | 24.983898 | 25.038336 | 24.658836 | NaN | 26.156054 | 26.229439 |  |
|  | C9YPV0 |  | 25.697104 | 25.233961 | 24.424576 | 25.462296 | 25.660957 | 25.518124 | 25.068007 | 24.799168 |  |
|  | C9YPV9 |  | 25.824154 | 25.507545 | 25.454378 | 26.007229 | 26.095329 | 24.529814 | 26.484545 | 26.486141 |  |
|  | C9YPW1 |  | 24.528683 | NaN | 26.274265 | 26.314789 | 24.578077 | NaN | 26.362188 | 24.116167 |  |
|  | C9YPW4 |  | 24.580494 | 25.090956 | 25.068417 | 24.360516 | 24.043350 | 23.805891 | 24.762129 | 25.259939 |  |
|  | C9YPW5 |  | NaN | NaN | NaN | 23.071005 | NaN | NaN | NaN | NaN |  |
|  | C9YPW7 |  | NaN | 23.527650 | 23.601536 | 22.975630 | NaN | 24.097641 | NaN | NaN |  |
|  | C9YPX2 |  | 27.594629 | 27.222324 | 27.127506 | 27.083143 | 27.511715 | 28.191736 | 27.081621 | 27.354137 |  |
|  | C9YPX8 |  | 30.016245 | 30.141752 | 29.742895 | 29.839241 | 28.942041 | 30.769354 | 30.166985 | 29.739454 |  |
|  | C9YPY4 |  | 23.874151 | 24.746119 | 22.492440 | 24.133438 | 23.756540 | NaN | 23.194529 | 24.346698 |  |
|  | C9YPY8 |  | 22.078680 | 23.415391 | NaN | 23.001959 | 22.351921 | NaN | 22.829622 | 22.358597 |  |
|  | C9YPY9 |  | 25.712273 | 26.522110 | 26.106274 | 25.793524 | 25.520523 | 26.297558 | 25.788355 | 25.784740 |  |
|  | C9YPZ0 |  | 28.987556 | 29.003460 | 28.899803 | 28.774704 | 28.517807 | 28.926855 | 28.754841 | 28.749735 |  |
|  | C9YPZ1 |  | 28.381088 | 29.049654 | 28.592812 | 27.971256 | 28.566271 | 28.610344 | 27.745415 | 28.161348 |  |
|  | C9YQ07 |  | 29.422119 | 29.965267 | 29.230272 | 29.000427 | 28.884018 | 31.007248 | 29.572411 | 29.545744 |  |
|  | C9YQ10 |  | 28.914648 | 28.178120 | 27.568991 | 27.648216 | 28.154654 | 27.254759 | 28.037844 | 27.519459 |  |
|  | C9YQ11 |  | 29.162285 | 30.007582 | 29.746984 | 30.161469 | 29.681555 | 30.389565 | 29.801840 | 29.585936 |  |
|  | C9YQ13 |  | NaN | NaN | 27.504534 | NaN | 28.199291 | 26.898506 | 28.166195 | 27.404057 |  |
|  | C9YQ14 |  | 24.916431 | 24.024536 | 25.722523 | 23.831903 | 25.593119 | 25.256775 | 25.331730 | 25.768553 |  |
|  | C9YQ15 |  | 32.129398 | 30.701572 | 31.189899 | 22.441843 | 31.785423 | 30.494192 | 31.853632 | 31.690083 |  |
|  | C9YQ17 |  | 25.925650 | 26.943914 | 30.527386 | 29.288021 | 36.712032 | 31.856863 | 37.398315 | 37.035881 |  |
|  | C9YQ18 |  | NaN | NaN | 25.751794 | NaN | 25.430777 | 27.971420 | 26.407141 | 26.460859 |  |
|  | C9YQ19 |  | 27.386814 | 26.902422 | 27.667768 | 26.939997 | 27.300686 | NaN | 27.114361 | 27.105396 |  |
|  | C9YQ20 |  | 25.947842 | 30.784721 | 26.050177 | 26.967962 | 25.871031 | NaN | 25.689472 | 26.062479 |  |
|  | C9YQ21 |  | 28.112720 | 33.383682 | 28.562782 | 29.021177 | 28.615932 | 22.420572 | 28.565435 | 28.767746 |  |
|  | C9YQ23 |  | 23.301035 | 25.768553 | 23.160542 | 22.639020 | 22.939108 | 25.048970 | 23.282181 | 22.992584 |  |
|  | C9YQ25 |  | 25.051298 | 25.161269 | 25.706488 | 24.939615 | 25.756617 | NaN | 24.625557 | 25.025658 |  |
|  | C9YQ43 |  | 31.306335 | 30.619381 | 30.694698 | 30.812300 | 31.248360 | 29.913136 | 30.392843 | 30.446972 |  |
|  | C9YQ47 |  | NaN | NaN | 22.762352 | NaN | 22.719399 | NaN | NaN | NaN |  |
|  | C9YQ50 |  | 26.605549 | 25.345671 | 23.387060 | 25.383703 | 26.021194 | 27.488535 | 24.923340 | 24.935844 |  |
|  | C9YQ54 |  | 26.776934 | 25.389524 | 26.508121 | 25.868677 | 26.332550 | 24.711721 | 25.582821 | 25.807030 |  |
|  | C9YQ56 |  | 30.237061 | 29.863605 | 29.879322 | 29.700298 | 29.896919 | 29.294739 | 28.203312 | 28.333344 |  |
|  | C9YQ57 |  | NaN | NaN | NaN | NaN | NaN | 24.734072 | NaN | NaN |  |
|  | C9YQ66 |  | 22.784645 | NaN | 23.475931 | 22.471174 | 23.495947 | NaN | 23.201496 | 23.566046 |  |
|  | C9YQ67 |  | NaN | NaN | NaN | 23.995331 | NaN | NaN | 24.140085 | 24.367064 |  |
|  | C9YQ68 |  | 25.894442 | 23.072272 | 25.677988 | 25.863441 | 25.175200 | NaN | 23.717432 | 23.659924 |  |
|  | C9YQ71 |  | NaN | NaN | 23.502396 | 25.837088 | NaN | 23.887778 | 22.293797 | NaN |  |
|  | C9YQ73 |  | 24.140193 | 24.413513 | 23.950550 | 24.728788 | 24.407433 | 24.145927 | 24.113148 | 24.312229 |  |
|  | C9YQ97 |  | 25.281712 | 27.605337 | 27.091541 | 26.986959 | 26.696573 | NaN | 26.926035 | 26.722080 |  |
|  | C9YQ98 |  | 25.868725 | 28.102245 | 27.402676 | 27.238268 | 27.320620 | NaN | 27.274355 | 27.153736 |  |
|  | C9YQC3 |  | 24.418476 | 23.808626 | 24.533443 | 24.280367 | 24.800156 | 24.314928 | 23.852530 | 23.840862 |  |
|  | C9YQC4 |  | 23.825222 | 23.505791 | 23.776857 | 23.844601 | 23.886881 | 23.796200 | 23.411499 | 23.346586 |  |
|  | C9YQD2 |  | 26.145130 | 27.765533 | 26.809803 | 26.774296 | 26.461218 | 25.631647 | 25.311501 | 25.726686 |  |
|  | C9YQD5 |  | 27.387060 | 28.295792 | 28.159426 | 27.544216 | 27.126818 | 25.288206 | 26.978365 | 28.126768 |  |
|  | C9YQD7 |  | NaN | NaN | NaN | 26.847328 | NaN | NaN | NaN | NaN |  |
|  | C9YQF0 |  | NaN | 24.280155 | 25.238705 | 25.308969 | 25.702133 | 25.400282 | 24.718426 | 24.921026 |  |
|  | C9YQF1 |  | NaN | NaN | 23.585316 | 23.802427 | 23.770521 | NaN | 22.997072 | 23.068153 |  |
|  | C9YQF2 |  | NaN | 23.184696 | NaN | 23.989365 | 23.502974 | NaN | 23.876034 | 23.206709 |  |
|  | C9YQG9 |  | 26.777060 | 27.879585 | 27.363359 | 28.179258 | 27.531109 | 27.549807 | 28.467388 | 28.057602 |  |
|  | C9YQH8 |  | 25.825439 | 24.436697 | 26.655127 | 26.174381 | 26.595055 | 22.723528 | 27.635403 | 27.807226 |  |
|  | C9YQI7 |  | 30.166506 | 31.670464 | 31.017639 | 30.849165 | 30.892366 | 33.653629 | 31.235691 | 31.377928 |  |
|  | C9YQK1 |  | 25.074268 | 25.606199 | 25.412415 | 25.904284 | 26.085272 | 24.831711 | 25.548397 | 24.984333 |  |
|  | C9YQK3 |  | 27.073675 | 26.747656 | 27.129965 | 27.256380 | 27.213823 | 27.920877 | 27.528057 | 27.519234 |  |
|  | C9YQK9 |  | 25.911869 | 25.053909 | 25.257351 | 25.727257 | 24.821043 | 25.734253 | 24.859276 | 24.999981 |  |
|  | C9YQL1 |  | 25.324265 | 25.357498 | 24.376236 | 25.456854 | 24.965212 | 25.035402 | 26.370659 | 26.028210 |  |
|  | C9YQN1 |  | 22.935944 | 24.446890 | 22.708261 | 23.578192 | 22.617889 | 22.496777 | 22.206844 | 22.195905 |  |
|  | C9YQN5 |  | 23.628018 | 26.605267 | 21.296336 | 20.787081 | 21.228281 | 21.233097 | NaN | 20.599596 |  |
|  | C9YQN9 |  | 25.448938 | 26.145617 | 26.646639 | 26.273483 | 25.774271 | 26.992588 | 25.972111 | 26.179401 |  |
|  | C9YQP3 |  | 25.786861 | 26.039570 | NaN | 24.268709 | 25.890596 | NaN | 25.357096 | 25.502607 |  |
|  | C9YQS4 |  | 23.986156 | 24.460890 | 23.989885 | 24.725880 | 24.137665 | NaN | 23.836727 | 23.896984 |  |
|  | C9YQW5 |  | NaN | NaN | NaN | NaN | NaN | 26.934158 | 26.250698 | 26.524893 |  |
|  | C9YR03 |  | 27.116642 | 26.367630 | 26.809927 | 26.230337 | 26.063261 | 27.241728 | 26.143087 | 26.591921 |  |
|  | C9YR08 |  | 32.488194 | 32.350765 | 31.312355 | 32.138279 | 32.389797 | 32.316566 | 32.007214 | 32.311409 |  |
|  | C9YR09 |  | 25.791563 | 29.292833 | 28.876368 | 28.444626 | 27.988693 | 27.369030 | 27.380882 | 27.778439 |  |
|  | C9YR10 |  | 26.571337 | 26.253641 | 26.898506 | 26.951271 | 26.501865 | 26.616247 | 26.922863 | 26.696573 |  |
|  | C9YR11 |  | 25.699699 | 25.149071 | 26.722342 | 25.486710 | 25.767948 | 26.159328 | 25.461920 | 25.648668 |  |
|  | C9YR12 |  | 32.475761 | 31.768053 | 32.456112 | 32.277943 | 32.404968 | 31.968395 | 31.920609 | 32.169853 |  |
|  | C9YR20 |  | 24.228540 | 24.473145 | 24.401976 | 26.912394 | 25.073002 | 25.227989 | 23.420727 | 23.617246 |  |
|  | C9YR21 |  | 22.921761 | 22.591972 | 24.407755 | 27.318380 | 23.763548 | 23.026908 | 21.743410 | 21.940056 |  |
|  | C9YR27 |  | 25.590380 | 25.687391 | 26.519999 | 26.573303 | 25.430426 | NaN | 26.523113 | 26.805136 |  |
|  | C9YR30 |  | 26.992697 | 26.230099 | 26.529203 | 27.129080 | 26.847328 | 26.837015 | 26.752386 | 26.705961 |  |
|  | C9YR47 |  | 24.567032 | 25.163654 | 23.260979 | 23.501545 | 24.808086 | NaN | 23.834929 | 23.736836 |  |
|  | C9YR63 |  | 23.598326 | 25.235094 | 25.067883 | 25.362154 | 23.944584 | 23.956210 | 23.543568 | 24.061758 |  |
|  | C9YR65 |  | 27.542004 | 27.609499 | 27.212154 | 27.710501 | 27.517733 | 27.997065 | 27.971090 | 27.822077 |  |
|  | C9YR67 |  | 29.610451 | 30.346359 | 30.049860 | 29.979374 | 30.482700 | 30.170450 | 30.107243 | 30.369633 |  |
|  | C9YR69 |  | NaN | NaN | NaN | NaN | NaN | NaN | NaN | 23.977623 |  |
|  | C9YR73 |  | 26.856855 | 27.813845 | 27.270884 | 27.016058 | 27.033596 | 28.434679 | 26.841822 | 27.276485 |  |
|  | C9YR74 |  | 29.438690 | 29.489607 | 30.384634 | 29.964441 | 30.017439 | 29.449297 | 30.051289 | 30.528784 |  |
|  | C9YR77 |  | NaN | NaN | NaN | 24.906006 | NaN | NaN | NaN | NaN |  |
|  | C9YR79 |  | 32.619797 | 31.084677 | 31.935999 | 32.228771 | 32.147495 | 33.289532 | 32.243546 | 31.941711 |  |
|  | C9YR83 |  | 26.464602 | 27.045414 | 27.406816 | 27.303476 | 25.930576 | 28.084360 | 26.578882 | 26.964771 |  |
|  | C9YR85 |  | 28.151512 | 28.395788 | 28.966835 | 28.586348 | 27.554060 | 29.252481 | 28.520958 | 28.723122 |  |
|  | C9YR87 |  | 24.821869 | 23.687020 | 25.392672 | 25.446701 | 24.651793 | 25.392736 | 25.164076 | 26.063261 |  |
|  | C9YR92 |  | NaN | NaN | 23.463324 | NaN | 23.909355 | 24.201500 | 24.215195 | 24.853052 |  |
|  | C9YR96 |  | 22.339831 | 22.532433 | NaN | 22.363913 | NaN | NaN | 22.140835 | 23.062302 |  |
|  | C9YRB2 |  | 29.105795 | 28.811182 | 27.608654 | 29.372894 | 29.063837 | 29.486982 | 28.342783 | 28.642616 |  |
|  | C9YRC4 |  | 28.178690 | 28.583265 | 28.094564 | 30.458263 | 28.584843 | 28.224627 | 30.477112 | 29.856619 |  |
|  | C9YRC5 |  | NaN | 24.305216 | 22.812332 | 23.651628 | NaN | NaN | 23.592749 | 23.407173 |  |
|  | C9YRC7 |  | 27.964109 | 28.803350 | 27.538677 | 28.042078 | 27.785128 | 29.320791 | 28.012440 | 27.735376 |  |
|  | C9YRC8 |  | 31.505594 | 31.700373 | 31.067343 | 31.726025 | 31.734690 | 31.523325 | 32.018566 | 31.521360 |  |
|  | C9YRD2 |  | 24.064720 | 25.060440 | 24.459265 | 24.915884 | 23.773441 | 24.957218 | 23.926239 | 24.366064 |  |
|  | C9YRE8 |  | 30.680349 | 31.353107 | 30.283833 | 30.916485 | 30.340748 | 31.149223 | 30.651642 | 30.362440 |  |
|  | C9YRE9 |  | 24.685814 | 24.597870 | 25.425856 | 24.835909 | 25.125261 | 24.631842 | 24.876474 | 25.137547 |  |
|  | C9YRF3 |  | 28.716858 | 29.582441 | 28.558664 | 28.578955 | 28.560999 | 24.503853 | 28.056154 | 28.131781 |  |
|  | C9YRF4 |  | NaN | 22.981983 | 21.688250 | 22.459265 | 21.543449 | NaN | NaN | 21.441061 |  |
|  | C9YRF7 |  | 26.343351 | 24.553152 | 26.726503 | 26.721689 | 25.990599 | 25.260368 | 26.300808 | 25.888090 |  |
|  | C9YRF8 |  | 24.990622 | 23.776457 | 24.491218 | 25.121393 | 24.417896 | NaN | 24.745811 | 24.663403 |  |
|  | C9YRH8 |  | NaN | NaN | NaN | 22.804338 | NaN | 24.049015 | NaN | NaN |  |
|  | C9YRI2 |  | 27.013079 | 26.419392 | 26.523249 | 26.806980 | 26.496115 | NaN | NaN | NaN |  |
|  | C9YRJ8 |  | 28.005871 | 29.672497 | 28.889019 | 28.591707 | 27.212061 | 27.350433 | 28.272221 | 28.681372 |  |
|  | C9YRK2 |  | 24.518152 | 24.913010 | 24.929312 | 24.770721 | 24.686777 | 25.335449 | 24.312159 | 24.566103 |  |
|  | C9YRK6 |  | NaN | NaN | 21.514702 | 21.981234 | 21.734969 | 22.190027 | 21.606807 | 21.522770 |  |
|  | C9YRK9 |  | 23.572091 | NaN | 23.844122 | 22.965502 | 23.864763 | 23.111637 | 22.870306 | 24.091600 |  |
|  | C9YRL0 |  | 23.979805 | NaN | 23.169653 | 22.087997 | 23.665682 | 23.266026 | 22.279943 | 23.113482 |  |
|  | C9YRL3 |  | 26.297733 | 23.962082 | 22.578953 | NaN | NaN | NaN | NaN | 22.772440 |  |
|  | C9YRL8 |  | NaN | 25.542917 | 23.395725 | 23.146221 | 24.122972 | 24.000177 | 22.026716 | 22.110825 |  |
|  | C9YRM3 |  | 27.733826 | 27.462404 | 27.649862 | 27.024326 | 27.633186 | 27.746952 | 27.270973 | 27.150253 |  |
|  | C9YRM5 |  | 27.503473 | 27.070097 | 27.294653 | 26.963449 | 27.288328 | 26.916044 | 27.385580 | 27.519533 |  |
|  | C9YRP2 |  | 24.998217 | 24.457262 | 25.263954 | 25.441961 | 24.745043 | NaN | 24.776054 | 24.882317 |  |
|  | C9YRR0;C9YRQ9 |  | NaN | NaN | NaN | NaN | NaN | NaN | 28.016590 | 27.532522 |  |
|  | C9YRR7 |  | NaN | NaN | NaN | NaN | NaN | NaN | 26.304207 | 25.641218 |  |
|  | C9YRR8 |  | NaN | NaN | NaN | NaN | NaN | NaN | 25.613890 | 25.331287 |  |
|  | C9YRS3 |  | 23.300619 | 24.157537 | 22.696297 | 23.757252 | 23.177689 | NaN | 23.519293 | 22.923605 |  |
|  | C9YRS6 |  | 25.133595 | 25.693466 | 25.982920 | 26.067186 | 25.422491 | 25.305182 | 25.075779 | 25.041723 |  |
|  | C9YRS7 |  | NaN | NaN | NaN | NaN | NaN | NaN | 22.394510 | 22.526932 |  |
|  | C9YRT1 |  | 33.137043 | 31.390591 | 32.527176 | 31.604568 | 32.552361 | 33.585644 | 32.230263 | 32.439487 |  |
|  | C9YRT3 |  | 23.773715 | 23.717329 | 24.026094 | 22.849671 | 22.564512 | NaN | 23.078989 | 23.450104 |  |
|  | C9YRT5 |  | 24.244756 | 25.132811 | 24.112432 | 24.065243 | 24.424063 | NaN | 24.689271 | 23.774748 |  |
|  | C9YRT6 |  | 29.050303 | 29.631748 | 28.888758 | 28.988993 | 29.150496 | 28.791662 | 29.077145 | 28.702892 |  |
|  | C9YRT7 |  | 24.761267 | 25.112553 | 24.448248 | 24.311813 | 24.428925 | 24.139618 | 24.318035 | 24.129120 |  |
|  | C9YRT8 |  | 27.560488 | 28.105896 | 27.425425 | 27.260153 | 27.468632 | 27.012012 | 27.262396 | 27.049990 |  |
|  | C9YRT9 |  | 25.704298 | 27.117237 | 25.465162 | 26.205830 | 26.242599 | 26.661938 | 26.346127 | 25.305321 |  |
|  | C9YRU0 |  | 25.294424 | 26.889135 | 25.060852 | 25.171272 | 25.647736 | 26.005432 | 25.469982 | 25.063076 |  |
|  | C9YRU7 |  | 22.866428 | 23.817142 | NaN | 22.501497 | 23.077328 | NaN | 23.017701 | 23.045798 |  |
|  | C9YRU8 |  | 27.863428 | 27.827234 | 27.873554 | 27.732662 | 28.076328 | 28.369070 | 27.529547 | 27.597544 |  |
|  | C9YRV5 |  | 26.696838 | 31.427773 | 26.645382 | 24.796150 | 28.700514 | 28.981390 | 25.095631 | 24.978670 |  |
|  | C9YRW5 |  | 30.741497 | 31.321146 | 31.156656 | 31.141327 | 30.767540 | 31.646544 | 30.908205 | 30.846178 |  |
|  | C9YRW9 |  | 26.802549 | 27.594273 | 26.747528 | 26.877247 | 26.928522 | 27.726439 | 26.640860 | 26.951048 |  |
|  | C9YRY2 |  | NaN | NaN | NaN | NaN | NaN | NaN | 24.835571 | 24.837545 |  |
|  | C9YRY3 |  | 25.482592 | 24.415319 | 23.675907 | 26.172245 | 25.614788 | 24.174629 | 25.689178 | 24.922977 |  |
|  | C9YRZ0 |  | 29.017412 | 29.715532 | 30.008652 | 29.270817 | 29.139950 | 29.575226 | 29.953381 | 29.903255 |  |
|  | C9YRZ4 |  | NaN | 24.965168 | NaN | 25.019669 | NaN | NaN | NaN | NaN |  |
|  | C9YS06 |  | 23.110052 | NaN | 22.590900 | 22.720685 | 23.214304 | NaN | 23.127121 | 23.641079 |  |
|  | C9YS11 |  | 26.365179 | 25.957085 | 27.122282 | 26.611752 | 26.317516 | 27.096977 | 25.898623 | 26.172131 |  |
|  | C9YS22 |  | 25.830816 | 26.012758 | 25.770393 | 25.709251 | 25.685173 | 25.805382 | 25.772158 | 25.429979 |  |
|  | C9YS24 |  | 27.838099 | 28.147781 | 25.380503 | 26.392754 | 27.724163 | 26.813478 | 26.456667 | 27.115948 |  |
|  | C9YS26 |  | NaN | NaN | 22.601547 | NaN | NaN | NaN | NaN | 22.502663 |  |
|  | C9YS34 |  | 25.078060 | 24.655127 | 24.925968 | 25.035318 | 24.927551 | 24.840429 | 24.885950 | 25.370993 |  |
|  | C9YS39 |  | 27.118128 | 27.421497 | 28.430178 | 27.696970 | 26.377146 | 27.346634 | 27.680902 | 28.458225 |  |
|  | C9YS40 |  | 22.832464 | 24.388769 | 22.852568 | 23.518934 | 23.053959 | NaN | 23.565231 | 23.841629 |  |
|  | C9YS50 |  | NaN | NaN | 22.973162 | 26.264383 | 21.932182 | NaN | 22.962170 | 21.917288 |  |
|  | C9YS62 |  | 24.296070 | 23.246496 | 23.232512 | NaN | 23.599119 | 24.156303 | 23.093634 | 22.494418 |  |
|  | C9YS63 |  | 23.980503 | NaN | 24.342588 | NaN | NaN | 22.888443 | 22.627048 | 24.116379 |  |
|  | C9YS65 |  | 27.874025 | 26.549044 | 27.902767 | 28.072144 | 27.801502 | 28.750534 | 28.280340 | 28.083651 |  |
|  | C9YS68 |  | 26.704510 | 26.769133 | 26.685120 | 26.839300 | 26.950270 | 27.166721 | 26.504169 | 26.618910 |  |
|  | C9YS78 |  | 24.761925 | 24.871077 | 25.283409 | 23.574270 | 24.109884 | 23.897631 | 23.878113 | 25.267031 |  |
|  | C9YS79 |  | 23.771931 | 23.562206 | 24.293758 | 23.203203 | 23.020269 | 23.754095 | 23.216793 | 24.062746 |  |
|  | C9YS81 |  | 24.896429 | 28.346001 | 25.028210 | 24.383373 | 23.945299 | 25.725880 | 24.246773 | 23.918798 |  |
|  | C9YS85 |  | NaN | NaN | NaN | NaN | 22.435808 | 26.713453 | NaN | NaN |  |
|  | C9YS93 |  | NaN | NaN | NaN | NaN | NaN | NaN | 25.225822 | 24.864197 |  |
|  | C9YSE6 |  | 27.449631 | 27.355146 | 26.909191 | 27.479156 | 27.197792 | 27.530291 | 28.879469 | 29.077883 |  |
|  | C9YSF2 |  | 22.463760 | 22.499382 | 23.476425 | 22.554943 | 22.683460 | 22.742731 | 22.188580 | 22.477463 |  |
|  | C9YSF3 |  | 24.550629 | 23.810589 | 23.655691 | 24.030275 | 23.832098 | 25.165342 | 23.964552 | 24.378817 |  |
|  | C9YSF4 |  | 24.601556 | 25.208103 | 23.762634 | 25.734564 | 24.254726 | 25.001257 | 25.171082 | 24.440036 |  |
|  | C9YSF5 |  | 29.795259 | 30.069201 | 29.968842 | 30.133202 | 29.879761 | 30.626884 | 29.627354 | 29.942516 |  |
|  | C9YSF6 |  | 31.063040 | 29.538677 | 31.023930 | 30.740452 | 31.522108 | 31.096413 | 30.752544 | 30.693369 |  |
|  | C9YSF8 |  | 24.124710 | 25.444807 | 24.153292 | 24.835716 | 23.863251 | 23.902975 | 23.889391 | 23.910130 |  |
|  | C9YSF9 |  | 29.778908 | 30.219511 | 29.204689 | 29.531054 | 29.257481 | 29.733309 | 29.279610 | 28.835360 |  |
|  | C9YSG1 |  | 28.272264 | 28.464586 | 27.860531 | 27.918491 | 27.752514 | 28.598076 | 28.294521 | 28.289824 |  |
|  | C9YSG7 |  | 23.123523 | NaN | 23.068581 | 22.686136 | NaN | NaN | 22.284143 | 22.778704 |  |
|  |  |  |  |  |  |  |  |  |  |  |  |

Table S3: LFQ analysis of all quantifiable proteins depicted as log2 median protein LFQ intensities for each PCR-ribotype. NaN represent values not detected in the particular isolates.


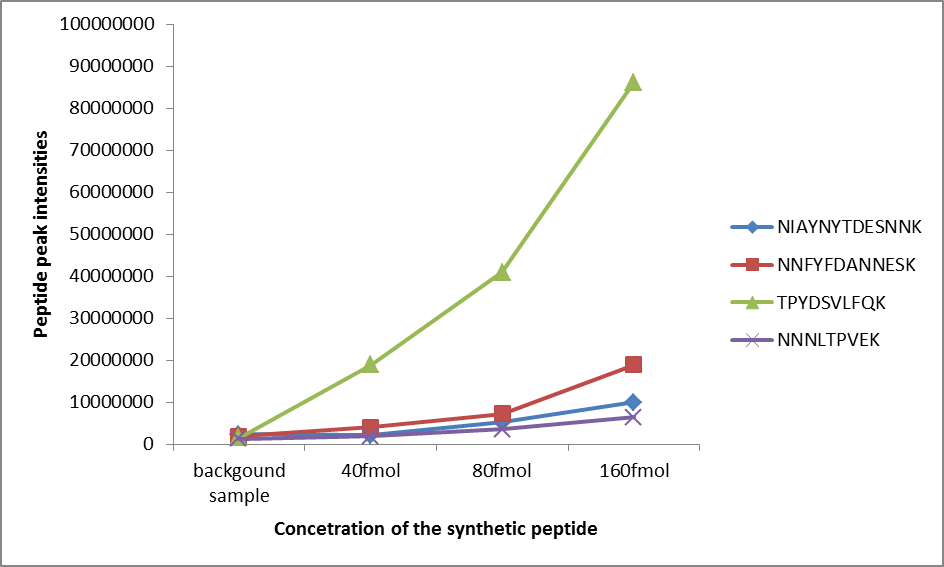


Figure S1: Peptide intensities of TcdA and TcdB synthetic peptides spiked into sample of the toxin negative strain (background sample)


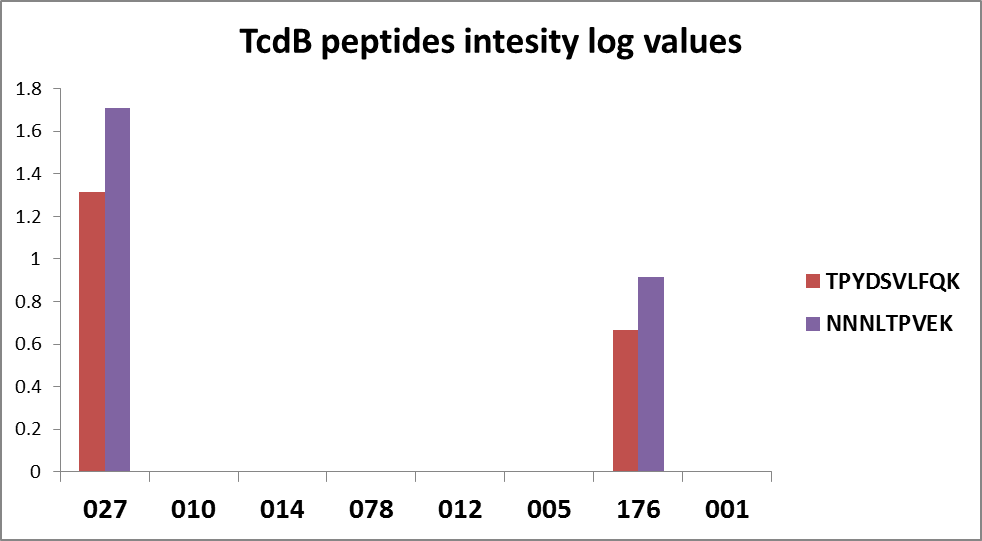


*
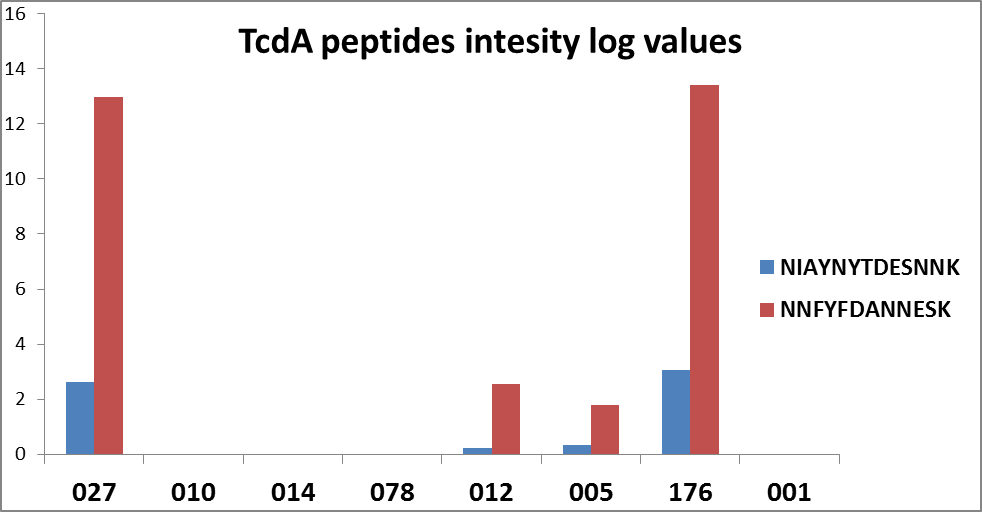
*

Figure S2: TcdA and TcdB levels depicted as Log value of ratio of light observed peptide intensity to its heavy labeled counterpart (40fmol spike).

Table S4: Mass spectrometry data and secretion prediction for analyzed proteins, ^1^Secretion mode of identified proteins: SigP = classically secreted protein: Signal P Score > 0.5; SecP = alternatively secreted protein: Secretome P Score > 0

| **Protein IDs** | **Gene name** | **Other name** | **Database (strain)** | **Sec^1^** | **Unique peptides** | **Sequence coverage (%)** | **Mol. weight (kDa)** | **Score** | **MS/MS Count** |
| --- | --- | --- | --- | --- | --- | --- | --- | --- | --- |
| **C9YJ37** | *tcdA* |  | R20291 | SecP | 103 | 50.8 | 308.31 | 323.31 | 481 |
| **C9YJ35** | *tcdB* |  | R20291 | SecP | 69 | 39.8 | 269.14 | 323.31 | 304 |
| **C9YPH7** | CDR20291_2491 | Binary toxin A | R20291 | SecP | 14 | 38.2 | 53.249 | 175.18 | 67 |
| **C9YPH8** | CDR20291_2492 | Binary toxin B | R20291 | SigP | 19 | 31.8 | 98.81 | 270.99 | 107 |
| **C9YK92** | CDR20291_0993 | Sigma-54 dependent regulatory protein | R20291 | SecP | 6 | 24.2 | 41.883 | 40.654 | 19 |
| **C9YLG4** | CDR20291_1416 | Nucleic acid zinc binding protein | R20291 | No | 7 | 35.3 | 27.573 | 121.58 | 30 |
| **C9YLL2** | CDR20291_1464 | Cell wall binding repeat 2 family | R20291 | SigP | 17 | 23.2 | 89.289 | 66.419 | 57 |
| **C9YRI2** | CDR20291_3199 | Putative nitroreductase | R20291 | No | 6 | 40.1 | 19.795 | 27.61 | 67 |
| **C9YQW5** | CDR20291_2983 | Abc-type Fe3+ transport protein | R20291 | SigP | 6 | 22.5 | 39 | 32.216 | 19 |
| **C9YI65** | *flgE* |  | R20291 | SecP | 14 | 46.5 | 34.632 | 224.78 | 128 |
| **C9YI79** | *flgG* |  | R20291 | SecP | 5 | 28.4 | 28.174 | 201.93 | 42 |
| **C9YI39** | *flgK* |  | R20291 | SecP | 22 | 43.8 | 47.966 | 242.21 | 140 |
| **C9YI40** | *flgL* |  | R20291 | SecP | 20 | 65.8 | 34.239 | 323.31 | 174 |
| **C9YI47** | *fliC* |  | R20291 | SecP | 17 | 59.2 | 34.374 | 323.31 | 482 |
| **C9YI45** | *fliD* |  | R20291 | SecP | 32 | 63.3 | 56.335 | 323.31 | 411 |
| **C9YI63** | *fliK* |  | R20291 | SecP | 8 | 21.9 | 46.128 | 48.163 | 35 |
| **C9YI80** | CDR20291_0273 | Flagellar basal body protein | R20291 | SecP | 9 | 46.6 | 27.987 | 215.02 | 75 |
| **C9YI56** | *flgC* |  | R20291 | SecP | 5 | 38.3 | 14.027 | 17.854 | 24 |
| **C9YI37** | *flgM* |  | R20291 | SecP | 3 | 36.3 | 10.376 | 19.98 | 22 |
| **C9YI69** | *fliL* |  | R20291 | No | 4 | 25.9 | 16.574 | 6.6329 | 12 |
| **C9YI57** | *fliE* |  | R20291 | No | 3 | 30.5 | 12.167 | 24.01 | 9 |
| **C9YI34** | CDR20291_0227 | Glycosyltransferase | R20291 | secP | 8 | 27.2 | 27.066 | 90.282 | 42 |
| **C9YQ56** | CDR20291_2721 | The Pro-Pro endopeptidase PPEP-1 | R20291 | SigP | 6 | 39.1 | 24.379 | 184.59 | 192 |
| **C9YQ57** | CDR20291_2722 | Collagen binding protein | R20291 | SigP | 6 | 6.5 | 108.98 | 20.265 | 13 |
| **Q17ZZ0** | CD630_32460 | Putative surface protein | 630 | SigP | 5 | 7.7 | 79.93 | 19.049 | 11 |
| **C9YLE4** | *hisZ* |  | R20291 | No | 13 | 43.9 | 37.077 | 195.75 | 193 |
| **C9YLE5** | *hisG* |  | R20291 | No | 15 | 72.6 | 23.491 | 118.34 | 192 |

|  |  | **Ribotype** |  | | | | | | |
| --- | --- | --- | --- | --- | --- | --- | --- | --- | --- |
| **PEPTIDE** | **PROTEIN ID** | **027** | **010** | **014** | **078** | **012** | **005** | **176** | **001** |
| NIAYNYTDESNNK+Label:13C(6)15N(2)(13) | TcdA | 377390953 | 494432678 | 558052681 | 502684513 | 447770311 | 475034980 | 424566272 | 577890907 |
| NIAYNYTDESNNK | TcdA | 3175415.54 | 9548.38 | 0 | 42110.704 | 284013.62 | 75588.734 | 9265275 | 16242.91 |
| light/heavy |  | 0.00841413 | 1.9312E-05 | 0 | 8.3772E-05 | 0.00063428 | 0.00015912 | 0.02182292 | 2.8107E-05 |
| ratio to PCR-ribotype 010 |  | 435.698989 | 1 | 0 | 4.33784938 | 32.8444009 | 8.23965357 | 1130.03084 | 1.45544383 |
| Log |  | 2.63918655 | 0 |  | 0 | 0.23456 | 0.3456 | 3.05309029 | 0 |
| NNFYFDANNESK+Label:13C(6)15N(2)(12) | TcdA | 278216918 | 461053418 | 473534559 | 458663521 | 388727914 | 404806238 | 287516364 | 313689003 |
| NNFYFDANNESK | TcdA | 5485912.47 | 0.000001 | 0 | 0 | 279670.543 | 260113.725 | 15905255.2 | 0 |
| light/heavy |  | 0.01971811 | 2.1689E-15 | 0 | 0 | 0.00071945 | 0.00064256 | 0.05531948 | 0 |
| ratio to PCR-ribotype 010 |  | 9.0911E+12 | 1 | 0 | 0 | 3.3171E+11 | 2.9626E+11 | 2.5505E+13 | 0 |
| Log |  | 12.9586166 | 0 |  |  | 2.567 | 1.7689 | 13.4066293 |  |
| TPYDSVLFQK+Label:13C(6)15N(2)(10) | TcdB | 351410165 | 443965684 | 481049434 | 438386106 | 350987278 | 328254195 | 243567043 | 278907262 |
| TPYDSVLFQK | TcdB | 43282174.4 | 31868125.1 | 11407942.6 | 26724070.6 | 68708989.6 | 37391658.9 | 80749312 | 41613001.4 |
| light/heavy |  | 0.12316711 | 0.0717806 | 0.0237147 | 0.06096012 | 0.1957592 | 0.11391068 | 0.33152807 | 0.14920014 |
| ratio to PCR-ribotype 010 |  | 1.71588287 | 1 | 0.33037754 | 0.84925618 | 2.72718799 | 1.58692842 | 4.61863025 | 2.07855781 |
| Log |  | 0.23448764 | 0 | 0 | 0 | 0 | 0 | 0.6645132 | 0 |
| NNNLTPVEK+Label:13C(6)15N(2)(9) | TcdB | 418670292 | 425545633 | 492908650 | 466710603 | 455437815 | 536769724 | 481007888 | 729876160 |
| NNNLTPVEK | TcdB | 5802937.96 | 1647493.06 | 1896512.52 | 1938882.59 | 2994156.67 | 3120816.95 | 15345933.1 | 3425460.82 |
| light/heavy |  | 0.0138604 | 0.00387148 | 0.00384759 | 0.00415436 | 0.00657424 | 0.00581407 | 0.0319037 | 0.00469321 |
| ratio to PCR-ribotype 010 |  | 3.58012612 | 1 | 0.99382935 | 1.07306588 | 1.69811844 | 1.50176794 | 8.24069133 | 1.21225049 |
| Log |  | 0.55389833 | 0 | 0 | 0 | 0 | 0 | 0.91596365 | 0 |

Table S5: Signal intensities and ratios of selected TcdA and TcdB peptides and their 40 fmol heavy counterparts
